# Microglia Polarization and Antiglioma Effects Fostered by Dual Cell Membrane-Coated Doxorubicin-Loaded Hexagonal Boron Nitride Nanoflakes

**DOI:** 10.1021/acsami.3c17097

**Published:** 2023-12-05

**Authors:** Özlem Şen, Melis Emanet, Martina Mazzuferi, Martina Bartolucci, Federico Catalano, Mirko Prato, Stefania Moscato, Attilio Marino, Daniele De Pasquale, Giammarino Pugliese, Francesco Bonaccorso, Vittorio Pellegrini, Antonio Esau Del Rio Castillo, Andrea Petretto, Gianni Ciofani

**Affiliations:** † Smart Bio-Interfaces, Istituto Italiano di Tecnologia, Viale Rinaldo Piaggio 34, Pontedera, Pisa 56025, Italy; ‡ Department of Mechanical & Aerospace Engineering, Politecnico di Torino, Corso Duca degli Abruzzi 24, Torino 10129, Italy; § Core Facilities-Clinical Proteomics and Metabolomics, IRCCS Istituto Giannina Gaslini, Via Gerolamo Gaslini 5, Genova 16147, Italy; ∥ Electron Microscopy Facility, Istituto Italiano di Tecnologia, Via Morego 30, Genova 16163, Italy; ⊥ Materials Characterization Facility, Istituto Italiano di Tecnologia, Via Morego 30, Genova 16163, Italy; # Department of Clinical and Experimental Medicine, University of Pisa, Via Roma 55, Pisa 56126, Italy; ∇ Nanochemistry, Istituto Italiano di Tecnologia, Via Morego 30, Genova 16163, Italy; ○ BeDimensional SPA, Lungotorrente Secca 30R, Genova 16163, Italy; ◆ Graphene Laboratories, Istituto Italiano di Tecnologia, Via Morego 30, Genova 16163, Italy

**Keywords:** glioblastoma multiforme, microglia, M2 polarization, hexagonal boron nitride nanoflakes, homotypic targeting, doxorubicin

## Abstract

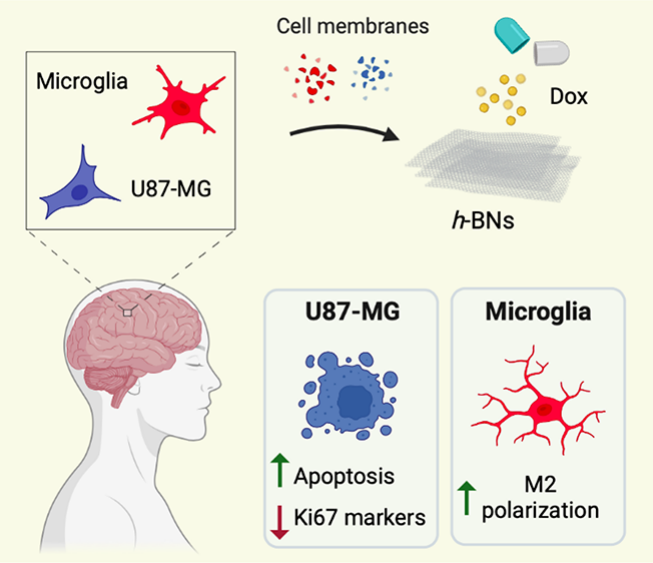

Microglial cells play a critical role in glioblastoma multiforme (GBM) progression, which is considered a highly malignant brain cancer. The activation of microglia can either promote or inhibit GBM growth depending on the stage of the tumor development and on the microenvironment conditions. The current treatments for GBM have limited efficacy; therefore, there is an urgent need to develop novel and efficient strategies for drug delivery and targeting: in this context, a promising strategy consists of using nanoplatforms. This study investigates the microglial response and the therapeutic efficacy of dual-cell membrane-coated and doxorubicin-loaded hexagonal boron nitride nanoflakes tested on human microglia and GBM cells. Obtained results show promising therapeutic effects on glioma cells and an M2 microglia polarization, which refers to a specific phenotype or activation state that is associated with anti-inflammatory and tissue repair functions, highlighted through proteomic analysis.

## 1 Introduction

Glioblastoma multiforme (GBM) is the most common and malignant type of brain cancer, accounting for 50% of all gliomas^1^ and derives from glial cells, the latter playing a variety of important functions including support to brain cells.^2^ Communications between cancer and non-neoplastic cells are considered critical in cancer progression,^3^ and among non-neoplastic cells, macrophages and microglial cells, which are 30–50% of the total mass of brain cancer, have a crucial impact on brain tumorigenesis and invasion.^4^

Microglia, innate immune cells, clean up debris in the central nervous system is required for a healthy environment; in this context, microglia activation and inflammatory response accompany many pathological diseases, including GBM.^5^ Microglia can become activated in response to various stimuli, such as injuries, infections, or tumor growth.^6^ Following activation, microglial cells show two main different polarizations: M1 (classically activated), pro-inflammatory and cytotoxic, and M2 (alternatively activated), anti-inflammatory, reactive oxygen species (ROS) scavenging, and immune-suppressive.^7^ In the context of GBM, microglia have been shown to play both pro-tumor and antitumor roles depending on the stage of tumor development and on the microenvironment.^8^ On one hand, microglia are known to be activated in response to glioblastoma cells, contributing to the growth and spread of the tumor by releasing pro-inflammatory molecules, *e.g*., cytokines and chemokines, which can stimulate the proliferation and migration of glioblastoma cells.^5^ On the other hand, microglia can provide a protective environment by secreting growth factors and creating a physical barrier that prevents the immune system from recognizing and attacking the tumor.^9^

Given the poor survival rate with the currently approved treatments for GBM, there is an urgent need to develop novel therapeutic strategies. Although current approaches involve surgical recession followed by chemotherapy and radiotherapy, chemotherapy is of limited efficacy because of the extensive infiltrative nature of GBM and the presence of the blood-brain barrier (BBB), hindering the passage of many drugs,^10^ which are thus required at high doses that might affect also healthy tissues. In recent years, nanoplatform-based technologies have been proposed to deliver therapeutic agents to specific targeted areas in a controlled manner, thus improving drug bioavailability.^11^ Hexagonal boron nitride (*h*-BN) nanostructures, analogously to graphene,^12^ have received extensive attention due to their biocompatibility, chemical stability, mechanical strength, and thermal conductivity.^13^ They have been used for several applications including composites, sensors, hydrogen storage, and recently as drug delivery systems.^13^*h*-BN have been investigated as a therapeutic component against several cancer types including glioblastoma,^14^ liver cancer,^15^ and ovarian cancer.^16^

This study describes the microglial response and therapeutic efficacy in GBM cells following the administration of dual cell membrane-coated and doxorubicin-loaded *h*-BN (Dox-CMC-*h*-BNs). The cell membrane coating enables the so-called “homotypic targeting”, i.e., the ability of cancer cells to recognize each other and interact owing to membrane molecules, mainly proteins. This approach overcomes the problems related to functionalization with ligands, *e.g.*, antibodies, proteins, or aptamers, in particular for highly heterogeneous cancers, *i.e*., GBM.^17^ After the coating of nanovectors was demonstrated, drug release profiles were tested under different experimental conditions. Then, their biocompatibility was tested on human microglia (HMC3), glioblastoma multiforme cells (U87-MG), and astrocyte cells, and the internalization was evaluated by flow cytometry, confocal microscopy, and Raman confocal microscopy. Apoptosis and proliferation assessments were carried out to test the therapeutic effects on U87-MG cells, while proteomic analysis was performed to evaluate the microglial response.

## 2 Experimental Section

### 2.1 Extraction of Cell Membranes and Nanocarrier Preparation

HMC3 (ATCC, CRL-3304) microglial cells were maintained in minimum essential medium (MEM, Gibco), supplemented with 1% v/v penicillin-streptomycin (P/S, Gibco) and 10% v/v fetal bovine serum (FBS, Gibco), while U87-MG cells (ATCC, HTB-14) were maintained in Dulbecco’s modified Eagle’s medium (DMEM, Gibco), supplemented with 1% v/v P/S, 1% v/v l-glutamine, and 10% v/v FBS. Cell membrane coating and doxorubicin (Dox) loading were achieved as previously described in a previous report.^18^ Briefly, HMC3 and U87-MG cells were separately cultured in 10 cm diameter Petri dishes, and when they reached 90% confluence, they were detached with a cell scraper and centrifuged at 600*g* for 5 min. The pellets were washed three times with phosphate buffer saline solution (PBS), resuspended in cold Milli-Q water, and then kept on ice. 5 × 10^6^ cells were disrupted with a high-pressure homogenizer with a 138 kPa homogenizing pressure. Thereafter, the samples were centrifuged at 10000*g* for 10 min; the supernatants were further centrifuged at 37000*g* for 60 min to collect cell membrane pellets, and finally resuspended in 1 mL of Milli-Q water.

To obtain cell membrane-coated *h*-BNs (CMC-*h*-BNs), 5 mg of *h*-BNs (BeDimensional, Italy)^19^ was added in 5 mL of cell membrane extract (derived from 25 × 10^6^ HMC3 cells and 25 × 10^6^ U87-MG cells). The dispersion was sonicated by using an ultrasonic tip (Fisherbrand Q125 Sonicator) for 20 min at 40% amplitude of power in an ice bath and then centrifuged at 10000*g* for 15 min at 4 °C. CMC-*h*-BNs were obtained after three rinses with Milli-Q water.

Dox-loaded *h*-BNs were prepared by sonicating 5 mg of *h*-BNs and 100 μg of Dox (Sigma-Aldrich) in 5 mL of Milli-Q water for 20 min at 40% amplitude of power in an ice bath and then kept at room temperature for 4 h. After incubation, Dox-loaded *h*-BNs were obtained by centrifuging at 10000*g* for 15 min and washing three times to remove nonattached Dox. To obtain Dox-loaded and cell-membrane-coated *h*-BNs (Dox-CMC-*h*-BNs), cell membrane coating was performed as described for bare CMC-*h*-BNs. A representation of the Dox-CMC-*h*-BNs preparation is depicted in Schema 1. Dox loading in Dox-CMC-*h*-BNs was indirectly calculated by measuring the free Dox present in the aqueous phase upon nanovector preparation. All the supernatants were collected after washing steps and were analyzed by a spectrofluorometer (Agilent Technologies Cary Eclipse; λ_ex_ = 470 nm; λ_em_ = 590 nm), exploiting a standard curve (Figure S1A).

**Scheme 1:**
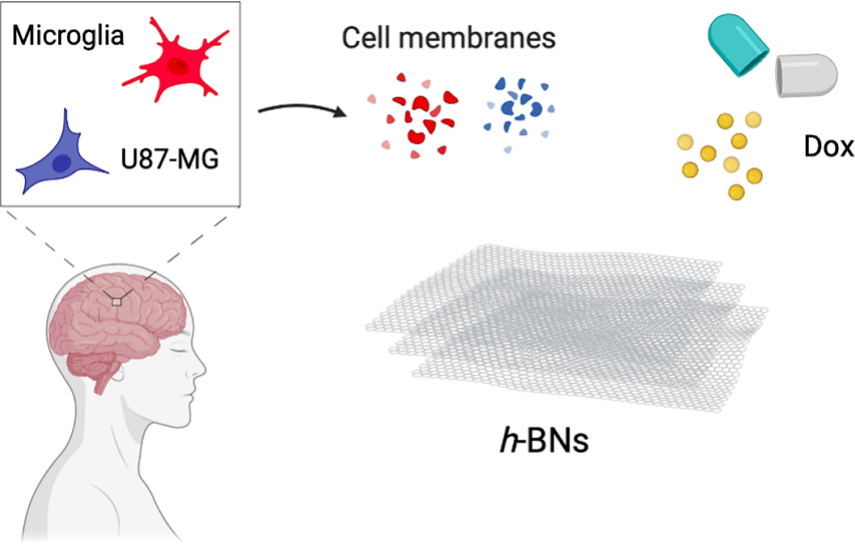
Preparation of Dox-CMC-*h*-BNs

### 2.2 Characterization of the Nanocarriers

Morphologic analyses of *h*-BNs, CMC-*h*-BNs, and Dox-CMC-*h*-BNs were performed by scanning electron microscopy (SEM) and transmission electron microscopy (TEM). For SEM analysis, a drop of the dispersion was cast on a silicon wafer and left to dry. Then, samples were gold-sputtered with 30 mA for 1 min with a Quorum Tech Q150RES Gold Sputter Coater and imaged with a Helios NanoLab 600i Dual BeamTM FIB/SEM FEI. For TEM analysis, each sample diluted into a concentration of 0.1 mg/mL was adsorbed for 60 s on plasma-cleaned pure carbon 300 mesh copper grids. After a washing step in Milli-Q water, the grids were stained with a solution of uranyl acetate (1% in distilled water) for 60 s. The resulting samples were then analyzed in bright-field mode using a JEOL JEM-1011 (JEOL, Japan) transmission electron microscope equipped with a thermionic source (W filament) operating at an acceleration voltage of 100 kV and with a Gatan Orius SC1000 series charge-coupled device (CCD) camera (4008 2672 active pixels).

Hydrodynamic diameter and ζ-potential measurements of *h*-BNs, CMC-*h*-BNs, and Dox-CMC-*h*-BNs were investigated in water at room temperature by using a Nano Z-Sizer 90 instrument (Malvern Instrument). The measurements are reported as the mean ± standard deviation of three different measurements with at least 10 runs for each of them. Long-term stability was assessed by storing the samples at 4 °C for 1 month and periodically performing dynamic light scattering (DLS) measurements.

The bicinchoninic acid assay (BCA, Thermo Scientific) was carried out to determine the amount of total proteins on the cell membrane-coated nanocarriers. Briefly, 25 μL of samples (100 μg/mL concentration) was added to 200 μL of working solution and incubated for 30 min at 37 °C. After incubation, the samples were centrifuged for 10 min at 7000*g* to eliminate residues, and the absorbance of the collected supernatants was measured at 560 nm by using a PerkinElmer Victor X3 microplate reader. A standard curve obtained with increasing concentrations of bovine serum albumin (BSA), was exploited (Figure S1B).

Thermogravimetric analysis (TGA) was conducted (TGA Q500-TA Instrument) under N_2_ over a temperature range from room temperature to 600 °C, at a heating rate of 10 °C min^–1^.

X-ray photoelectron spectroscopy (XPS) investigation was carried out with a Kratos Axis Ultra^DLD^ spectrometer, using a monochromatic Al Kα source, operated at 15 kV and 20 mA. The specimens were prepared by drop-casting a few microliters of concentrated dispersions of the samples onto silicon substrates. Wide scans were collected at a pass energy of 160 eV and an energy step of 1 eV; high-resolution scans were acquired at a pass energy of 20 eV and an energy step of 0.1 eV. A Kratos charge neutralization system was applied during data acquisition. The binding energy scale was calibrated by setting the main peak of the C 1s spectrum to 284.8 eV. Spectra were analyzed using the CasaXPS software (version 2.3.24).

Before western blot analysis, the cell membrane coating was removed from Dox-CMC-*h*-BNs using RIPA buffer (Sigma-Aldrich) and sonication. Briefly, 100 μL of RIPA buffer was added to 1 mg/mL Dox-CMC-*h*-BNs and the mixture sonicated at 8 W for 1 min inside an ice bath. Then, the solution was centrifuged at 5000*g* for 15 min. The protein content was determined from the obtained pellet using the BCA assay. Because of the low protein concentration, each sample was precipitated by adding 1 volume of 20% trichloroacetic acid (TCA, Sigma-Aldrich) to one volume of the sample, and the mixture was incubated in an ice bath for 1 h. After centrifugation at 11840*g* for 20 min at 4 °C, the supernatant was removed, and the precipitate was washed twice with 0.5 mL of cold acetone and centrifuged after each wash at 11840*g* for 20 min at 4 °C. The residual acetone was removed by allowing the pellet to dry at room temperature. To perform sodium dodecyl-sulfate polyacrylamide gel electrophoresis (SDS-PAGE), samples were resuspended in Laemmli Sample Buffer 1× with 2-mercaptoethanol (Bio-Rad); 10 μg/lane proteins were separated on a 4–20% polyacrylamide gel (BioRad) and transferred to polyvinylidene difluoride (PVDF) membranes using the Trans Turbo Blot system (BioRad). Membranes were blocked for 1 h with 5% dry fat milk in T-TBS (Tris Buffered Solution 1× containing Tween 0.1%) and then probed overnight at 4 °C with the following primary antibodies diluted in T-TBS: Rb antineural cadherin (N-cadherin) 1:500 (GeneTex, Cat No. GTX112733), Rb anticluster of differentiation 44 (CD44) 1:1000 (GeneTex, Cat No. GTX102111), Rb anti-β-catenin (β-catenin) 1:500 (GeneTex, Cat No. GTX101435), Rb antineuroplastin (NPTN) 1:500 (Abcam, Cat No. ab272652), and Rb anti Bcl-2 1:500 (GeneTex, Cat No. GTX100064). Secondary antirabbit HRP-conjugated antibody (BioRad) (1:2000 in 5% dry fat milk in T-TBS, 1 h) was used, and immunoreactive bands were detected by chemiluminescence (ECL clarity, BioRad), using a Chemi-Doc XR (BioRad). All reactions were performed at room temperature, unless otherwise specified. Image Lab Software (BioRad) was used to evaluate the correct molecular weights of the resulting bands.

### 2.3 Doxorubicin Release Tests

Dox cumulative release of Dox from Dox-CMC-*h*-BNs was tested at three different time points (4, 24, and 72 h) in four different experimental conditions (pH 4.5, pH 4.5 with 100 μM H_2_O_2_, pH 7.4, and pH 7.4 with 100 μM H_2_O_2_). 2 mL of Dox-CMC-*h*-BNs (1 mg/mL concentration) were incubated at 37 °C in a shaker at 150 rpm, protected from light exposure. Before analysis, the solutions were centrifuged at 10000*g* for 15 min, and the collected supernatants were analyzed through fluorescence spectroscopy (λ_ex_ = 470 nm; λ_em_ = 590 nm), by exploiting standard curves obtained for each specific experimental condition (Figure S1A). The pellets were redispersed with the same solvents and incubated in the same conditions described above for the following measurements.

### 2.4 Cell Viability Assays

The proliferation of U87-MG, HMC3, and astrocyte cells was assessed upon incubation with *h*-BNs, CMC-*h*-BNs, or Dox-CMC-*h*-BNs using the Quant-iT PicoGreen dsDNA assay kit (Invitrogen) following the manufacturer’s instructions. The cells were seeded at a density of 1 × 10^4^ cells/cm^2^ in 96-well plates and incubated overnight. Then, cultures were treated with *h*-BNs, CMC-*h*-BNs, or Dox-CMC-*h*-BNs at increasing concentrations (12.5–200 μg/mL) and with plain Dox (0.3–5 μg/mL, corresponding to 12.5–200 μg/mL of Dox-CMC-*h*-BNs), and further incubated for 24 and 72 h. At the end point, cells were rinsed and left under 250 μL of Milli-Q water before three freezing/thawing cycles between −80 °C and room temperature, to allow for complete cell lysis. After a centrifugation step to remove cellular debris, the dsDNA content was evaluated by mixing 100 μL of reaction buffer, 50 μL of cell lysate, and 150 μL of the PicoGreen reagent. After a 10 min incubation under shaking at room temperature, fluorescence emission (directly proportional to the dsDNA content and thus to cell number), has been measured by using the PerkinElmer Victor X3 microplate reader (λ_ex_ = 480 nm; λ_em_ = 520 nm); experiments have been performed in triplicate.

### 2.5 Internalization Studies

U87-MG cells were seeded in 24-well plates at a density of 1 × 10^4^ cells/cm^2^. After 24 h of incubation, Dox-CMC-*h*-BNs (25 μg/mL) were added, and cultures were incubated for an additional 24 and 72 h. After that, cultures were rinsed with PBS, treated with trypsin for 5 min at 37 °C, and centrifuged at 600*g* for 5 min. Each sample was collected in a flow cytometer tube by diluting with 200 μL of PBS. Finally, the cellular uptake of nanovectors was analyzed by a flow cytometer (Beckman Coulter CytoFLEX; λ_ex_ = 490 nm, λ_em_ = 565 nm).

For confocal microscopy imaging, HMC3, U87-MG, and astrocytes were seeded at 1 × 10^4^ cells/cm^2^ in μ-dishes (Ibidi) and incubated for 24 h. Then, they were treated with 25 μg/mL CMC-*h*-BNs for an additional 24 and 72 h. After incubation, the cells were fixed with 4% w/v paraformaldehyde (PFA, Sigma-Aldrich) at 4 °C for 20 min and washed three times with PBS. For the imaging of nuclei and *f*-actin, the cells were treated with Hoechst (1:1000 v/v, Invitrogen) and FITC-phalloidin (1:200 v/v, Sigma-Aldrich) at 37 °C for 45 min. Images were acquired with a confocal fluorescence microscope (C2 system Nikon).

To confirm the internalization of CMC-*h*-BNs by HMC3, U87-MG, and astrocytes, cells (density of 1 × 10^4^ cells/cm^2^) were seeded on Raman-grade calcium fluoride substrates (Crystran) and incubated for 24 h. Then, they were treated with 25 μg/mL CMC-*h*-BNs for 24 and 72 h. After incubation, the cultures were fixed with 100% v/v methanol for 5 min at −20 °C and analyzed by confocal Raman microscopy (Horiba’s LabRAM HR Evolution Confocal Raman Microscope equipped with 532 nm laser); a 10× objective was used to acquire the signals. As the control experiments, cells without CMC-*h*-BN treatment were considered. Pseudocolor maps were obtained according to the signal of *h*-BNs (in red, Raman shift range: 1350–1380 cm^–1^), and phenylalanine (in green, Raman shift range: 980–1020 cm^–1^), with a pixel intensity proportional to peak intensity (LabSpec 6 software).

### 2.6 Therapeutic Efficacy on Cancer Cells

To analyze the apoptotic behavior of the U87-MG cells upon incubation with Dox, CMC-*h*-BNs, or Dox-CMC-*h*-BNs, the cells were seeded at a density of 1 × 10^4^ cells/cm^2^ in 24-well plates and incubated overnight. Then, the cultures were treated with Dox (0.6 μg/mL), CMC-*h*-BNs (25 μg/mL), or Dox-CMC-*h*-BNs (25 μg/mL) and further incubated for 24 and 72 h. Thereafter, the cells were prepared for flow cytometry analysis (λ_ex_ = 485 nm; λ_em_ = 535 nm) by staining with annexin V-FITC (0.4 μg/mL in PBS, 15 min incubation in the dark at room temperature), and thereafter, percentages of alive and apoptotic cells were calculated from histograms using the area parameter. Three independent experiments were performed and expressed as mean values.

U87-MG cells were processed to monitor the immunofluorescence of the *K*_i_-67 marker upon incubation with Dox, CMC-*h*-BNs, or Dox-CMC-*h*-BNs by using the same treatment described above. At the end point, the cells were fixed with 4% (w/v) PFA with an incubation at 4 °C for 20 min and then washed with PBS three times. Thereafter, the cells were treated with Triton X-100 (0.1%, v/v, in PBS) for 30 min at room temperature. Following the removal of the Triton X-100 solution, the cells were treated with goat serum (10% (v/v) in PBS) for 30 min at room temperature to reduce nonspecific backgrounds. Following the goat serum removal, the cells were treated with a rabbit antihuman primary antibody against *K*_i_-67 protein (1:200; Sigma-Aldrich, in goat serum, 10% v/v) for 2 h at room temperature. Then, the cells were gently washed with PBS three times before staining with a fluorescein goat antirabbit IgG secondary antibody (1:200 v:v; Invitrogen, in goat serum, 10% v/v) and Hoechst (0.1% v/v in goat serum, 10% v/v) for 30 min at room temperature. Images have been acquired with a confocal fluorescence microscope; a minimum of 10 images have been obtained from different areas of the cell cultures, and the number of the cells, positive for nucleus and marker staining, were calculated and analyzed with the ImageJ software.

### 2.7 Proteomic Analysis

HMC3 cells were seeded (1 × 10^4^ cells/cm^2^) and left overnight to attach; thereafter, cultures have been treated for 24 h with Dox (0.6 μg/mL), CMC-*h*-BNs (25 μg/mL), or Dox-CMC-*h*-BNs (25 μg/mL); untreated cultures have been considered as well, as control. Samples were lysed, reduced, and alkylated in 50 mL of LYSE buffer (Preomics) at 95 °C for 10 min and sonicated with an Ultrasonic Processor UP200 St (Hielscher), 3 cycles of 30 s. 50 mg of lysate samples was digested by adding trypsin and LysC at a 1:50 and 1:100 ratio of enzyme:protein content, respectively, and incubated at 37 °C overnight. Digested samples were processed by the iST protocol.^20^

The resulting peptides were analyzed by a nano-UHPLC-MS/MS system using an Ultimate 3000 RSLC coupled to an Orbitrap Fusion Tribrid mass spectrometer (Thermo Scientific Instrument). Elution was performed with an EASY spray column (75 μm × 25 cm, 2 μm particle size, Thermo Scientific) at a flow rate of 400 nL/min using a linear gradient of 2–30% solution B (80% acetonitrile -ACN- and 20% H_2_O, including 5% dimethyl sulfoxide -DMSO- and 0.1% formic acid -FA-) in 46 min. MS analysis was performed in the DIA mode. Orbitrap detection was used for MS1 measurements at a resolving power of 120 K in a range between 375 and 1500 *m*/*z*, 300% automatic gain control (AGC) target, and 50 ms maximum injection time. Advanced Peak Determination was enabled for MS1 measurements. FAIMS compensation voltage (CV) was set to −50 at standard resolution and with a total carrier gas flow of 1.5 L/min. Precursors were selected for data-independent fragmentation with an isolation window width of 25 *m*/*z* in 24 windows ranging from 380 to 980 *m*/*z* and 2 *m*/*z* overlap. HCD collision energy was set to 30% and MS2 scans were acquired at a resolution of 15k, 1000% AGC target, and 22 ms maximum injection time (IT).

All DIA raw files were processed with Spectronaut version 16^21^ using a library-free approach (directDIA) under default settings. The library was generated against the UniProt Human database (release UP000005640_9606 February 2022). Carbamidomethylation was selected as a fixed modification, and methionine oxidation and N-terminal acetylation were selected as variable modifications. FDRs of PSMs and peptide/protein groups were set to 0.01. For quantification, Precursor Filtering was set to Identified (Qvalue) and MS2 was chosen as the quantity MS-level.

The Protein Quant Pivot Report generated by Spectronaut was statistically evaluated using Perseus software^22^ version 1.6.15.0. GO enrichment was obtained with the Web server ShinyGO (version 0.75).^23^ The mass spectrometry proteomics data have been deposited to the ProteomeXchange Consortium via the PRIDE^24^ partner repository with the data set identifier PXD043557.

### 2.8 Statistical Analysis

Statistical analysis was performed by using analysis of variance (ANOVA) followed by Bonferroni’s *post hoc* test. The significance was set at *p* < 0.05, and data were presented as the mean value ± standard deviation of three independent experiments.

## 3 Results

### 3.1 Characterization of Nanocarriers

**Figure 1:**
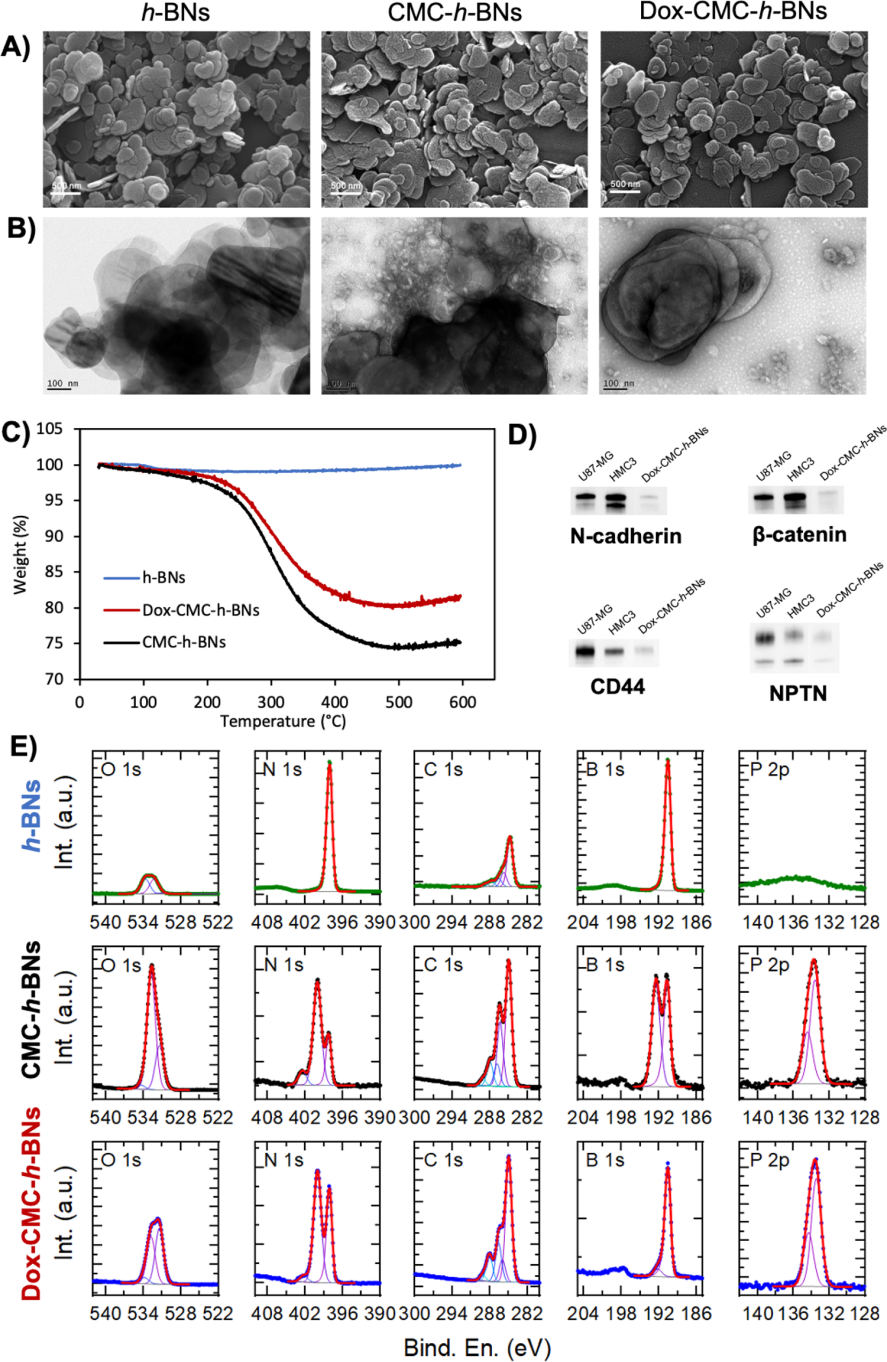
Representative (A) SEM and (B) TEM images of *h*-BNs, CMC-*h*-BNs, and Dox-CMC-*h*-BNs. (C) TGA results. (D) Western blot analysis for N-cadherin, beta-catenin, CD44, and NPTN proteins on Dox-CMC-*h*-BNs. (E) XPS analysis on *h*-BNs, CMC-*h*-BNs, and Dox-CMC-*h*-BNs.

Representative SEM and TEM images suggest the presence of typical discoidal nanoparticles, as depicted in Figure 1A,B, respectively. Elemental analysis was conducted by using scanning electron microscopy with energy-dispersive X-ray spectroscopy (SEM-EDS; Figure S2). Observed peaks resulted in boron at 0.19 keV, carbon at 0.29 keV, nitrogen at 0.39 keV, oxygen at 0.53 keV, and phosphorus at 2.05 keV. Carbon and phosphorus were exclusively observed in the CMC-*h*-BN sample, already giving a hint of a successful cell membrane coating.

The hydrodynamic size distributions of *h*-BNs, CMC-*h*-BNs, and Dox-CMC-*h*-BNs were assessed through DLS measurements (Table 1) and resulted in 431.9 ± 8.1, 301.1 ± 8.0, and 369.0 ± 7.7 nm, respectively, with a polydispersity index (PDI) of 0.305 ± 0.021, 0.286 ± 0.029, and 0.235 ± 0.025, respectively. The ζ-potential values of *h*-BNs, CMC-*h*-BNs, and Dox-CMC-*h*-BNs resulted in −19.5 ± 0.4, −26.4 ± 1.0, and −41.6 ± 1.3 mV, while the protein contents of CMC-*h*-BNs and Dox-CMC-*h*-BNs were 531.6 ± 54.7 and 527.2 ± 1.3 μg/mL, respectively.

**Table 1:** Physicochemical Characterization of *h*-BN, CMC-*h*-BN, and Dox-CMC-*h*-BN Aqueous Dispersions

	hydrodynamic diameter (nm)	polydispersity index (PDI)	ζ potential (mV)	protein concentration (μg/mL)
*h*-BNs	431.9 ± 8.1	0.305 ± 0.021	–19.5 ± 0.4	
CMC-*h*-BNs	301.1 ± 8.0	0.286 ± 0.029	–26.4 ± 1.0	531.6 ± 54.7
Dox-CMC-*h*-BNs	369.0 ± 7.7	0.235 ± 0.025	–41.6 ± 1.3	527.2 ± 1.3

The long-term stability of cell membrane-coated nanoparticles has been monitored using DLS and ζ-potential measurements for up to 30 days, as shown in Figure S3, and results showed that both CMC-*h*-BNs and Dox-CMC-*h*-BNs remained stable at 4 °C. Conversely, uncoated *h*-BNs show high polydispersity index (PDI) and hydrodynamic size values already at day 3, confirming significant aggregation and unsuitability for further biological applications.

Figure 1C shows the TGA results for nanoparticles before and after the cell membrane coating. The bare *h*-BNs started losing weight (≈1%) just at ≈100 °C due to the evaporation of water; conversely, weight losses of 24.8% and 18.3% for CMC-*h*-BNs and Dox-CMC-*h*-BNs at ≈300 °C are attributed to the cell membrane coating and to the presence of the drug.^18^

To demonstrate homotypic targeting through cell membrane coating, we assessed the expression of four different cell membrane markers involved in this process using western blotting, as depicted in Figure 1D: results show the presence of N-cadherin, β-catenin, CD44, and NPTN on the surface of Dox-CMC-*h*-BNs.

Figure 1E further assesses the cell membrane coating on the nanoparticles using XPS analysis. The presence of a boron B 1s peak at 190.5 ± 0.2 eV together with a nitrogen N 1s peak at 398.2 ± 0.2 eV in the spectra of all samples nanoparticles is consistent with the presence of the *h-*BN nanoflakes.^25^ The presence of oxygen O 1s and carbon C 1s signals (at approximately 532 and 285 eV, respectively) on the bare *h-*BNs is likely due to exposure to air.^26^ On the other two samples, instead, the presence of O 1s and C 1s signals, found at a higher extent with respect to bare *h-*BNs, together with the presence of phosphorus P 2p at 133.7 ± 0.2 eV, suggests the effective coating of *h-*BNs with cell membranes. It must be noted that the CMC-*h*-BN sample shows partial oxidation of the *h-*BNs, as indicated by the B 1s peak at 192.5 ± 0.2 eV, consistent with the presence of B_2_O_3_ or borates in the sample^25^; the loading of Dox induced the loss of these oxidized species.

Table 2 reports the quantification of the percentage of B, N, C, O, and P elements in all of the considered nanoparticles, while XPS wide-scan spectra of the samples are depicted in Figure S4.

**Table 2:** Quantification of % Elements in *h*-BNs, CMC-*h*-BNs, and Dox-CMC-*h*-BNs

	B (%)	N (%)	C (%)	O (%)	P (%)
*h*-BNs	44.0	43.6	7.8	4.6	0
CMC-*h*-BNs	5.8	5.9	64.9	22.6	0.8
Dox-CMC-*h*-BNs	7.4	12.4	64.7	14.6	0.8

**Figure 2:**
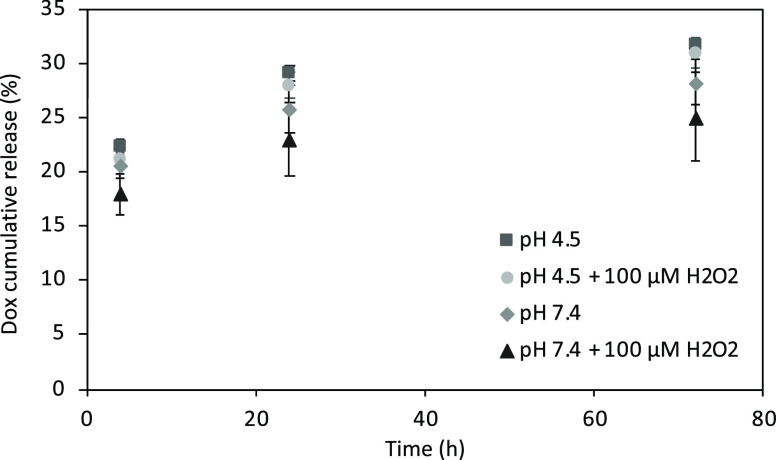
Cumulative Dox release profile (in %) from Dox-CMCs-*h*-BNs at increasing time points (4, 24, and 72 h) and under different treatments (at pH 4.5, pH 4.5 + 100 μM H_2_O_2_, pH 7.4, and pH 7.4 + 100 μM H_2_O_2_).

Concerning drug loading, 1 mg/mL Dox-CMC-*h*-BNs resulted in carrying 22.8 ± 0.1 μg/mL of Dox, thus resulting in an entrapment efficiency of 91.1 ± 0.4%. The cumulative Dox released from Dox-CMC-*h*-BNs at different time points (4, 24, and 72 h) and at different conditions (pH 4.5, pH 4.5 with 100 μM H_2_O_2_, pH 7.4, and pH 7.4 with 100 μM H_2_O_2_) is reported in Figure 2. At the end point, 32.9 ± 0.5% Dox was released at pH 4.5, 32.0 ± 1.3% at pH 4.5 with 100 μM H_2_O_2_, 28.3 ± 2.1% at pH 7.4, and 25.1 ± 4.1% at pH 7.4 with 100 μM H_2_O_2_, suggesting slow, progressive, and slightly increased release at acidic pH values.

### 3.2 Cell Viability

**Figure 3:**
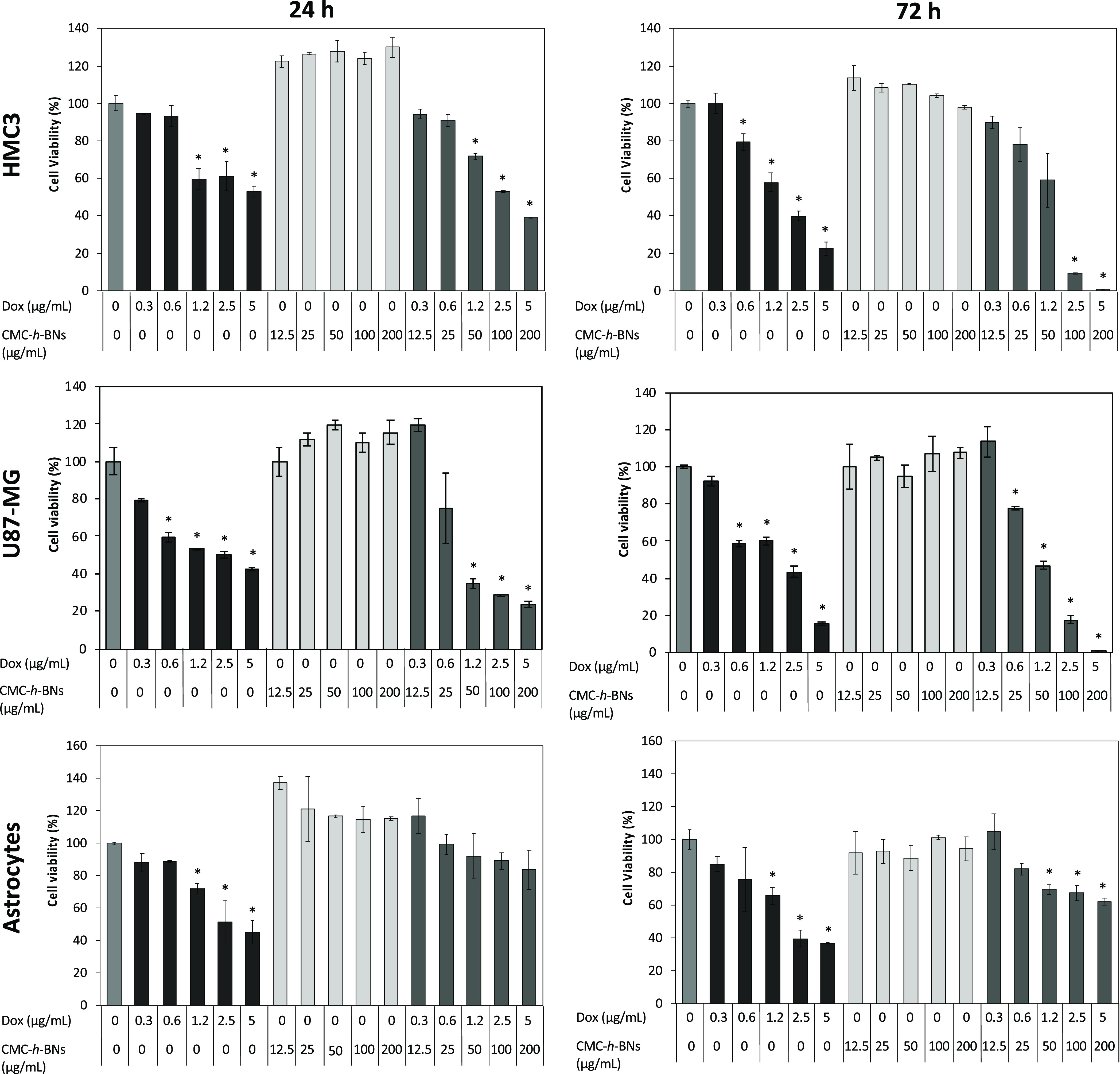
Biocompatibility studies on HMC3, U87-MG, and astrocytes treated with Dox, CMC-*h*-BNs, and Dox-CMC-*h*-BNs for 24 and 72 h. The values are presented as the mean ± standard deviation of 3 different measurements (* *p* < 0.05).

The cytocompatibility following treatment with Dox, CMC-*h*-BNs, or Dox-CMC-*h*-BNs was evaluated on HMC3, U87-MG, and astrocytes by the Picogreen assay, to analyze cell proliferation after 24 and 72 h of incubation. As shown in Figure 3, Dox significantly reduced the proliferation of HMC3, U87-MG, and astrocytes at 1.2, 2.5, and 5 μg/mL (corresponding to 50, 100, and 200 μg/mL of Dox-CMC-*h*-BNs); moreover, a reduction was also highlighted at 0.6 μg/mL (corresponding to 25 μg/mL of Dox-CMC-*h*-BNs) in HMC3 and U87-MG cells. On the contrary, cell viability was not significantly affected at any concentration of CMC-*h*-BNs. The proliferation of HMC3 and U87-MG cells treated with Dox-CMC-*h*-BNs was eventually significantly reduced at high concentrations (50, 100, and 200 μg/mL) at both time points (24 and 72 h); however, astrocyte proliferation was significantly affected just after 72 h of incubation.

### 3.3 Internalization Studies

**Figure 4:**
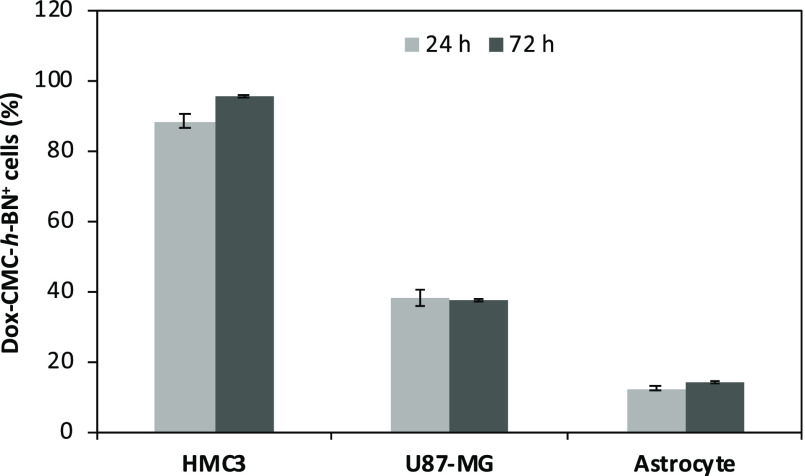
Flow cytometry quantification of the percentage of Dox-CMC-*h*-BN-positive cells on HMC3, U87-MG, and astrocyte cultures after 24 and 72 h of incubation.

Flow cytometry analysis was conducted to quantitatively investigate the percentage of Dox-CMC-*h*-BN-positive cells (i.e., that exhibit a detectable level of a specific marker or molecule of interest) in HMC3, U87-MG, and astrocyte cultures after incubation for 24 and 72 h (Figure 4). The highest extent of Dox-CMC-*h*-BN-positive cells was observed in HMC3 cultures, reaching 88.6 ± 2.1% and 95.8 ± 0.6% at 24 and 72 h, respectively, while in U87-MG cultures, 38.7 ± 2.4% and 37.8 ± 0.4%, and in astrocytes, just 12.6 ± 0.6% and 14.4 ± 0.4%. Representative flow cytometry plots are reported in Figure S5.

**Figure 5:**
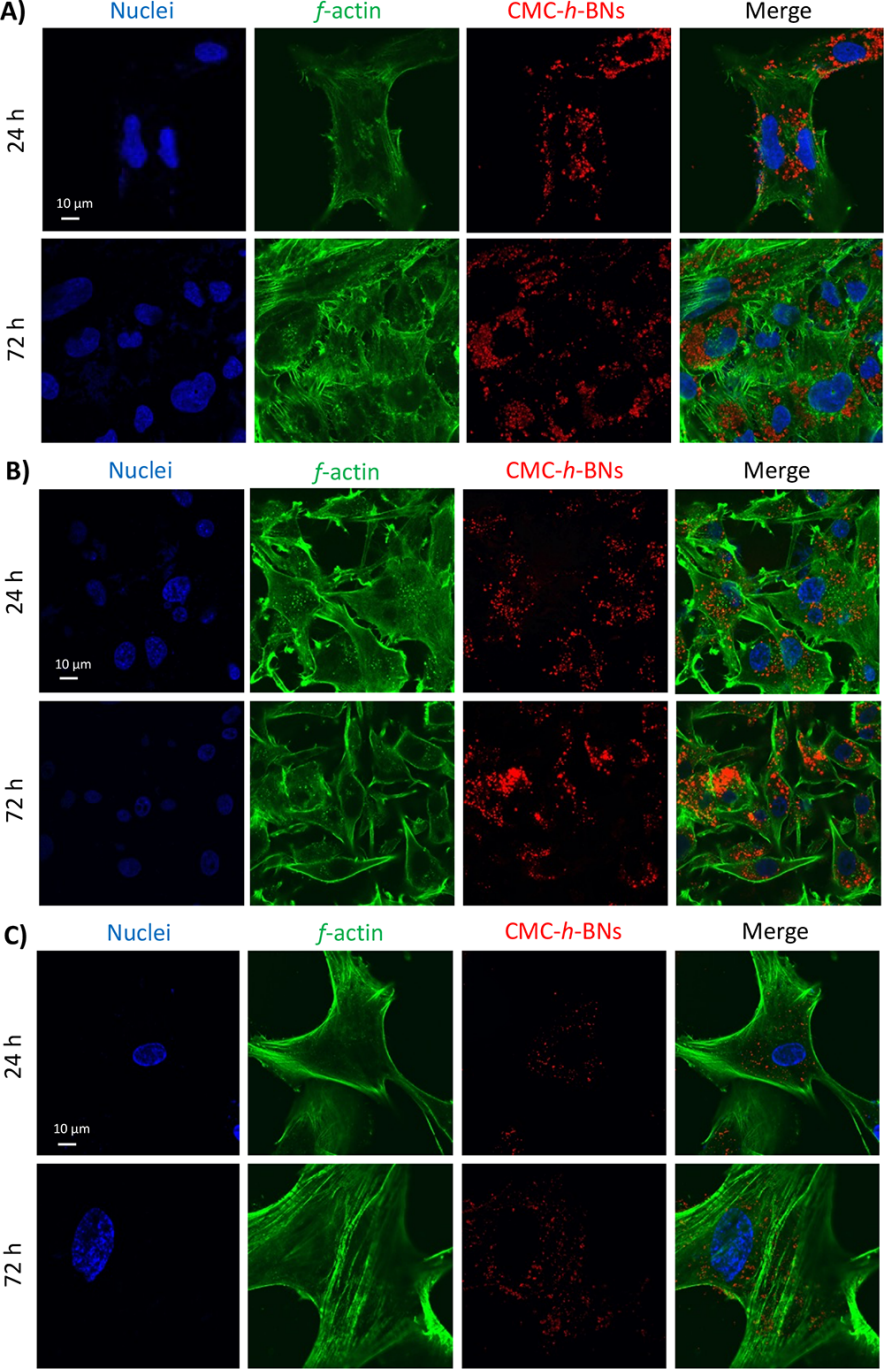
Representative confocal images of (A) HMC3, (B) U87-MG, and (C) astrocytes treated with Dox-CMC-*h*-BNs (red) for 24 and 72 h. Nuclei (blue) and *f*-actin (green) are also shown.

The cellular localization of Dox-CMC-*h*-BNs was qualitatively assessed using confocal microscopy in HMC3, U87-MG, and astrocytes after incubation for 24 and 72 h of incubation. Representative images (Dox-CMC-*h*-BNs in red, *f*-actin in green, and nuclei in blue) are reported in Figure 5 (3D rendering in Figure S6) and show that nanoparticles were found to be primarily located in the cytoplasm and distributed around the perinuclear region of the cells. A lower uptake extent was found in astrocytes, coherently to the flow cytometry data.

**Figure 6:**
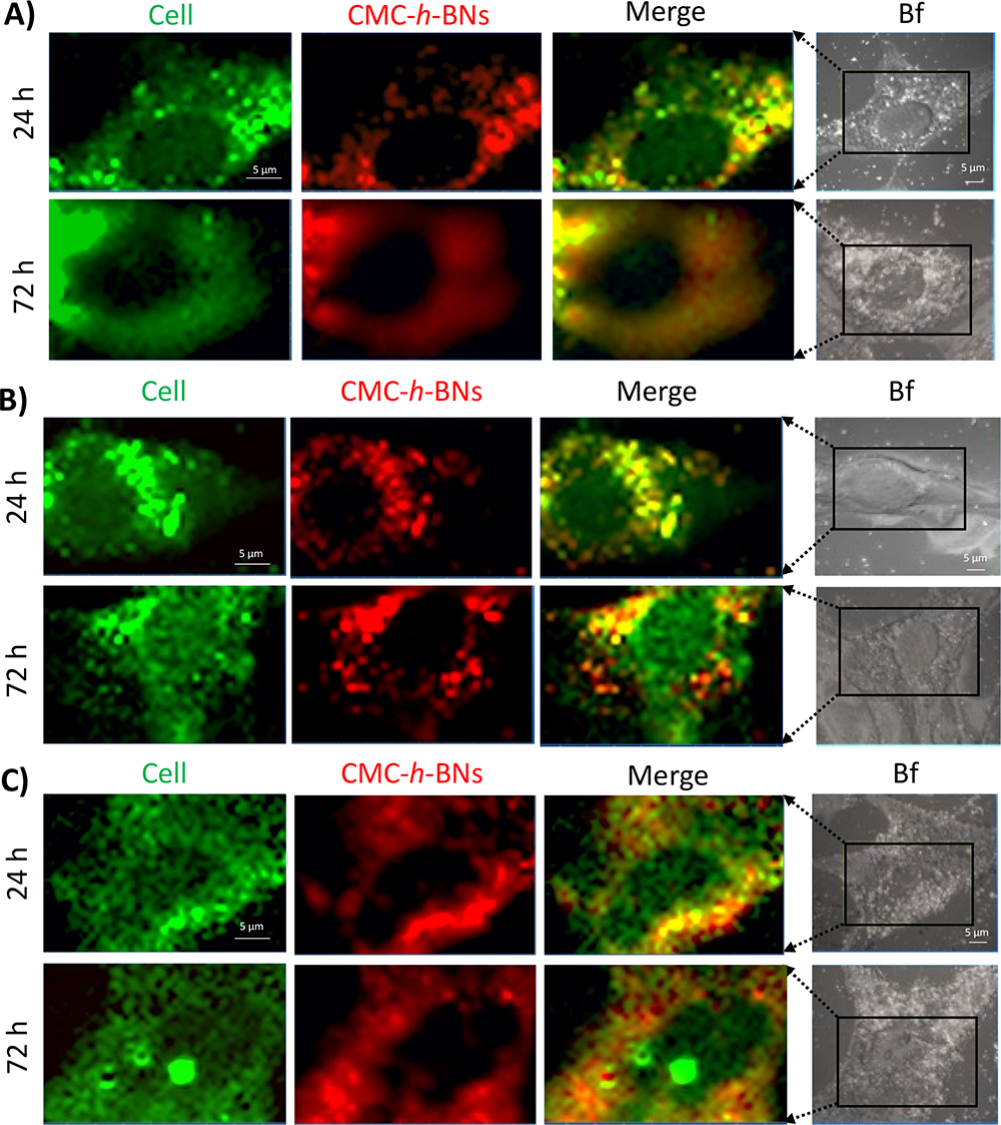
Confocal Raman imaging of (A) HMC3, (B) U87-MG, and (C) astrocytes treated with CMC-*h*-BNs for 24 and 72 h. Signal maps were obtained according to the signal of *h*-BNs (in red, Raman shift range: 1350–1380 cm^–1^) and of phenylalanine (in green, Raman shift range: 980–1020 cm^–1^).

To further analyze nanoparticle internalization, confocal Raman microscopy was exploited as a complementary tool for label-free investigation. Representative images of HMC3, U87-MG, and astrocytes were shown in Figure 6A–C, respectively, while representative signals from *h*-BNs and phenylalanine are shown in Figure S7. The range and the related map indicating the signal originating from *h*-BNs are depicted in red (Raman shift range: 1350–1380 cm^–1^), while the range and the related map indicating the signal from cells are in green (Raman shift range: 980–1020 cm^–1^). Results confirm an internalization of nanoparticles spread in the cytoplasm; imaging and spectra of HMC3, U87-MG, and astrocytes without nanoparticle treatment (as a control) are reported for comparison in Figure S7.

### 3.4 Therapeutic Efficacy on Cancer Cells

**Figure 7:**
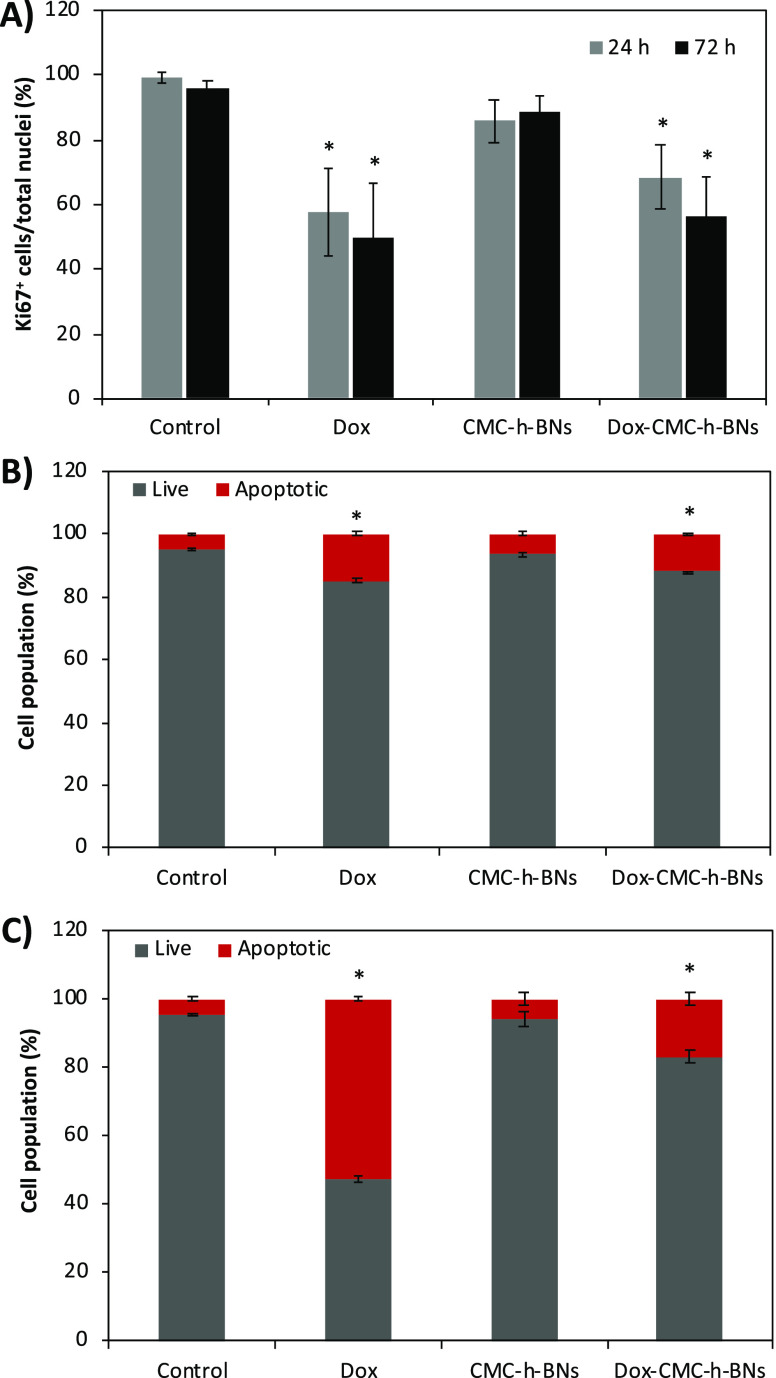
(A) Immunofluorescence analysis on Dox-, CMC-*h*-BNs-, and Dox-CMC-*h*-BNs-treated U87-MG cells for *K*_i_-67 marker after 24 and 72 h of incubation (* *p* < 0.05). Flow cytometry analysis of apoptotic and live cells treated with Dox, CMC-*h*-BNs, and Dox-CMC-*h*-BNs after (B) 24 h and (C) 72 h.

*K*_i_-67 is widely used as a biomarker to assess the proliferative activity of tumor cells^27^; this proliferation marker was analyzed in U87-MG cells following treatment with Dox, CMC-*h*-BNs, or Dox-CMC-*h*-BNs for 24 and 72 h. In Figures S8 and S9, the fluorescence signal stemming from *K*_i_-67 expression was evaluated to quantitatively assess the cell proliferation activity. As shown in Figure 7A, the analysis of the images shows that Dox- and Dox-CMC-*h*-BNs-treated cells significantly reduced the *K*_i_-67 marker expression, converse to the CMC-*h*-BN treatment, suggesting a reduced proliferation activity.

**Figure 8:**
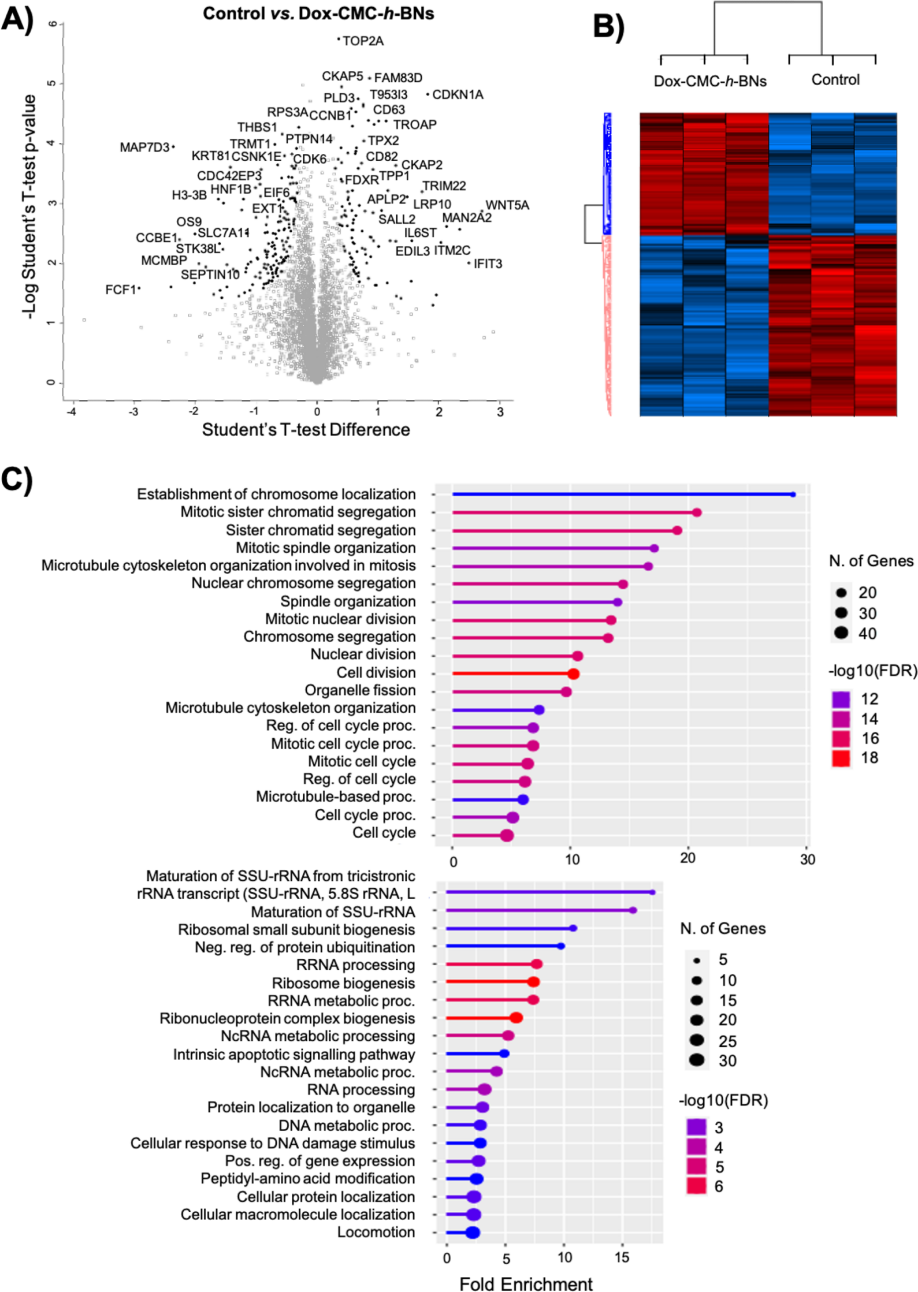
Proteomic analysis. (A) Volcano plot and (B) heatmap showing over- and underexpression of proteins for Dox-CMC-*h*-BNs-treated HMC3 cells with respect to control cultures. (C) Complete list of the gene ontology terms related to the Dox-CMC-*h*-BN treatment.

Apoptotic phenomena following the treatment with Dox, CMC-*h*-BNs, or Dox-CMC-*h*-BNs were evaluated at 24 and 72 h of incubation, as respectively shown in Figures 8B,C. The treatment with the Dox induced apoptosis in 15.7 and 52.7% of cells, while the treatment with Dox-CMC-*h*-BNs induced 11.9 and 16.9% of cells at 24 and 72 h of incubation, respectively. The significant increment in apoptosis is attributed to the exposure of the cells to the free Dox and to the Dox content in the Dox-CMC-*h*-BNs since CMC-*h*-BNs did not cause a significant increment of apoptotic cells with respect to the control group. Representative flow cytometry scatter plots of apoptosis experiments are reported in Figure S10.

### 3.5 Microglial Response

Proteomic analysis was carried out on HMC3 cells treated with Dox-CMC-*h*-BNs (Figure 8) to investigate the microglial response in terms of M1 and M2 polarization after 24 h of incubation. Proteomics allows to investigate the global changes in protein expression, post-translational modifications, and protein–protein interactions associated with microglial activation.^28^ M1 and M2 activation are two distinct polarization states of microglia, representing their functional phenotypes, which show classical pro-inflammatory (M1) and anti-inflammatory (M2) activities.^29^ As depicted in Figure 8A, a significant overexpression (10-fold, *p* < 0.05) was detected for cyclin-dependent kinase inhibitor 1 (CDKN1A), while trophinin-associated protein (TROAP) was 7-fold overexpressed (*p* < 0.05). In addition, significant overexpression (6-fold, *p* < 0.05) was found for the family with sequence similarity 83 member D (FAM83D), a cluster of differentiation 63 (CD63), cytoskeleton-associated protein 2 (CKAP2), hyaluronan mediated motility receptor (HMMR), tripartite motif containing 22 (TRIM22), and Wnt family member 5A (WNT5A) proteins. Significant under-expression (9-fold, *p* < 0.05) was instead found for microtubule-associated protein 7 domain-containing protein 3 (MAP7D3), while 6-fold under-expression was found for keratin type II cuticular Hb1 (KRT81). It was also found a 5-fold significant under-expression for histone H3 (H3–3B), mono-ADP ribosylhydrolase 1 (MACROD1), cell division control protein 42 homologue effector protein 3 (CDC42EP3), lysine acetyltransferase 8 regulatory nonspecific lethal complex subunit 2 (KANSL2), and hepatocyte nuclear factor 1-beta (HNF1B). A heatmap reporting all the overexpressed (in red) and underexpressed (in blue) proteins after being treated with Dox-CMC-*h*-BNs is depicted in Figure 8B with respect to untreated control cultures.

Gene ontology (GO) analysis indicated significant overexpression of proteins involved in “establishment of chromosome localization”, “mitotic sister chromatid segregation”, “sister chromatid segregation”, “mitotic spindle organization”, “microtubule cytoskeleton organization involved in mitosis”, “nuclear chromosome segregation”, “spindle organization”, “mitotic nuclear division”, “chromosome segregation”, “nuclear division”, and “cell division”. The complete list of the significantly changed GO terms has been reported in Figure 8C.

Proteomic analysis was also performed on cultures treated with Dox and CMC-*h*-BNs, as a control (Figure S11). A significant overexpression (6-fold, *p* < 0.05) of CDKN1A, TROAP, FAM83D, CKAP2, HMMR, TRIM22, and WNT5A and underexpression (5-fold, *p* < 0.05) of HNF1B, CDC42EP3, and microtubule-associated protein 7 domain containing 3 (MAP7D3) were found for cultures treated with Dox, while a significant overexpression (6-fold, *p* < 0.05) for CD63 and WNT5A and underexpression (5-fold, *p* < 0.05) for H3–3B were found for cultures treated with CMC-*h*-BNs.

## 4 Discussion

Glioblastoma multiforme is characterized by the rapid and uncontrolled growth of glial cells, which are the cells that provide support and protection for neurons in the brain.^30^ Standard treatments involve a combination of surgery, radiation therapy, and chemotherapy; however, the prognosis for GBM is poor, with a median survival time of around 15 months.^31^ Because BBB, tumor heterogeneity, and drug resistance over time limit the treatments, high doses of drugs can cause significant side effects and toxicity, making it challenging to find a balance between effective treatment and patient quality of life. To overcome these challenges, nanomaterial-based systems are proposed to enhance GBM tumor targeting, mainly by promoting drug crossing through the BBB and because of an improved targeting due to the permeability and retention (EPR) effect.^32^

Hexagonal boron nitride nanostructures have been investigated as a potential drug delivery system due to their unique properties, including high surface area, biocompatibility, and ability to be easily loaded with drugs.^33^ Additionally, the surface hydrophobicity of *h*-BNs makes them easy to interact with hydrophobic drugs (via hydrophobic interactions).^14^ Further to their largely documented biocompatibility, the selection of *h*-BNs as nanovectors for the delivery of therapeutic agents is mainly due to their versatility: *h*-BN nanostructures can be easily functionalized and engineered to carry therapeutic or diagnostic payloads, making them a very efficient theranostic nanoplatform.^34^ In our study, Dox was used as a chemotherapeutic agent still scarcely exploited to treat central nervous system tumors, mainly because of its poor ability to penetrate the BBB and its quick removal from brain tissue through active transport.^35^ These issues can be mitigated by an appropriate loading in “Trojan horses” nanostructures. Among the latter, limited research is currently available on the metabolism and elimination of *h*-BN nanostructures, specifically in the brain. However, it can be hypothesized that they can undergo various processes, including phagocytosis by immune cells, transport through the lymphatic system, and degradation. The latter may occur through enzymatic or chemical processes within the brain.^36^

Despite the potential benefits of nanomaterial-based drug delivery systems, nanoparticles are often cleared by the immune system and present poor targeting abilities. In this regard, the use of cell membrane-based strategies allows them to interact with biological systems naturally and effectively, coupled with good biocompatibility, strong targeting ability, long circulation time, and immune evasion.^37^ In our study, we used HMC3 and U87-MG cell membranes for *h*-BN stabilization in aqueous environments: the hydrophobic fatty acid chains interact with the hydrophobic side walls of *h*-BNs, while the hydrophilic phosphate chains interact with water, thus stabilizing the nanostructures.^18^

The decrease in hydrodynamic size measured by DLS after cell membrane coating of the nanoparticles can be attributed to several factors, such as surface charge modification, redistribution of biomolecules, and aggregation reduction.^38^ When nanoparticles are coated with cell membranes, they acquire an additional layer of lipids, proteins, and other biomolecules. This coating can alter the surface properties of the nanoparticles, making them more stable and less prone to aggregation or agglomeration. As a result, DLS may detect smaller hydrodynamic sizes because the coated nanoparticles are more dispersed and less likely to clump together.^39^ Furthermore, cell membranes often carry a net negative charge due to the presence of various proteins and lipids: coating nanoparticles with cell membranes can result in a change in the overall surface charge of the particles. If the plain nanoparticles had a tendency to repel each other due to electrostatic forces, the addition of a negatively charged cell membrane coating may further disperse the nanoparticles and lead to a smaller hydrodynamic size.^40^ Uncoated nanoparticles might have a higher propensity to aggregate in the solution, leading to larger apparent hydrodynamic sizes when measured by DLS: the addition of a cell membrane coating can act as a stabilizing agent, preventing or reducing nanoparticle aggregation and, consequently, reducing the measured hydrodynamic size.^41^

Thermogravimetric analysis was performed to evaluate the cell membrane amount coated on the *h*-BNs and to calculate the weight percentage of the coating (*h*-BNs are thermally stable up to 1000 °C^34^). Doxorubicin, when loaded onto *h*-BNs, contributes to the overall weight of the sample. In a thermogravimetric analysis, the weight loss is typically measured along sample heating, and any component that decomposes, evaporates, or undergoes chemical reactions contributes to this weight loss.^42^ If doxorubicin is present, its weight can offset some of the weight loss; furthermore, it may interact with the surface of the *h*-BNs, forming bonds or complexes: these interactions can change the thermal behavior of the drug and how it decomposes during the TGA, affecting the weight loss pattern.

EDS and XPS spectra confirmed the organic layer after the cell membrane coating. To further characterize the functionalization, four different cell membrane proteins (N-cadherin, β-catenin, CD44, and NPTN) were selected for western blotting analysis. N-cadherin, a member of the calcium-dependent adhesion molecule family of cadherins, plays a critical role in homotypic and heterotypic cell–cell interactions,^43^ while β-catenin is involved in intercellular adhesion.^44^ CD44, a cell surface adhesion receptor (transmembrane glycoprotein), is highly expressed in many cancer cells.^45^ Neuroplastin, a transmembrane protein of the immunoglobulin superfamily, belongs to a cell-adhesion molecule family.^46^

Considering the glioblastoma heterogeneity, translated into a differential expression of membrane-associated surface antigens/proteins,^47^ using GBM cell membranes for homotypic interaction is considered an improved approach for targeting, as already reported in the literature.^48^ The targeting of microglia and U87-MG cells was achieved through dual-cell membrane coating and verified by flow cytometry and confocal microscopy analysis. One of the most important advantages of homotypic targeting includes increased specificity and selectivity, as the treatment can be designed to target only specific cells while sparing healthy cells. This can result in reduced side effects and toxicity with respect to nonspecific treatments.

The cell viability results indicated that Dox shows a significant toxic effect on each cell line at both time points; in the case of loading on CMC-*h*-BNs, it does not show a significant toxic effect on astrocytes at 24 h of incubation. Moreover, a lower reduction of the astrocyte viability with respect to HMC3 and U87-MG cells in the case of exposure to Dox-CMC-*h*-BNs has been attributed to a lower cellular internalization by these cells.

The *K*_i_-67 protein is present just in the nucleus of cycling cells and is variably expressed in terms of quantity and topographic distribution according to the various phases of the cell cycle. *K*_i_-67 is widely used to estimate the potential proliferation of a tumor cell population; in many tumors, the percentage of *K*_i_-67-positive cells is correlated with parameters reflecting tumor aggressiveness or progression.^49^ In our study, the *K*_i_-67 reduction in the Dox and Dox-CMC-*h*-BN-treated U87-MG cells has been attributed to an inhibited proliferation of these cells. Since the Dox molecules naturally intercalate into the DNA double helix structure, they block the cell cycle at S or G2/M phase.^50^ A study performed on breast cancer patients indeed indicated the *K*_i_-67 reduction in case the cell cycle was arrested at the G2/M phase.^51^

Apoptosis, a programmed cell death, plays an essential role in both physiological and pathological processes in many human tissues since the defects in the regulation of this pathway potentially lead to many diseases, including glioblastoma.^52^ Observed increased apoptosis can be attributed to the Dox intercalation in the DNA, which stimulates p53 tumor suppressor protein expression and leads to programmed cell death.^53^

Glioma cells can prompt microglia to secrete various types of molecules that encourage the growth and advancement of the tumor, although the precise molecular mechanisms underlying these phenomena are still largely unclear.^54^ In this study, GBM cell membranes were used to observe the microglial response in the presence of cell membrane proteins. Proteomics have shown significantly modulated proteins associated with the Dox-CMC-*h*-BNs treatment on microglia cells: for example, cyclin-dependent kinase inhibitor 1 and TROAP were significantly overexpressed (10-fold and 7-fold, respectively). The former acts as a regulator of cell cycle progression at G1 by suppressing the function of cyclin–cyclin-dependent kinase complexes.^55^ Transcriptomic profiling of microglia shows that CDKN1A overexpression suggests cell proliferation;^56^ in another study, overexpression of CDKN1A was attributed to the activation of various biological pathways such as DNA damage response, cell cycle re-entry, and NFκB signaling.^57^ Trophinin-associated protein, a cytoplasmic protein, plays a role in microtubular cytoskeleton regulation, spindle assembly during mitosis, and cell cycle progression. Overexpression of TROAP was reported in glioblastoma cells resulting in promoting cell proliferation via the Wnt/β-Catenin signaling pathway,^58^ while in hepatocellular carcinoma, TROAP overexpression led cells to accumulate in the G1/S phase and to enhanced cell proliferation through direct interaction with dual specificity tyrosine phosphorylation regulated kinase 1 A/B (DYRK1A/B).^59^ The overexpression of CDKN1A and TROAP might be attributed to M2 polarization, where microglial cells increase proliferation and are involved in immune responses, tissue repair, and tissue remodeling.^60^

Microtubule-associated protein 7 domain-containing protein 3 and KRT81 were significantly underexpressed on microglia cells after being treated with Dox-CMC-*h*-BNs. Microtubule-associated protein 7 domain-containing protein 3 enhances the assembly and stability of microtubules.^61^ The downregulation of MAP7 and MAP7D1 was demonstrated to increase phosphorylation of p53, with a strong G1 arrest and the impairment of DNA repair mechanisms.^62^ Noteworthily, it was reported that the alteration of DNA damage repair mechanisms through overexpression of midkine (MDK) mediated by p53 in GBM promotes M2 polarization of microglia.^63^ Keratin type II cuticular Hb1 is highly expressed in many types of human cancers and is also involved in carcinogenesis.^64^ Although the role of keratin remains unclear in the CNS or neurodegenerative diseases, a study highlighted that HMC3 cells passively secrete KRT1, KRT5, and KRT9, while this secretion is not affected by TNF-α-induced inflammation.^65^ However, further studies will be necessary to fully understand the impact of the KRT81 protein in microglia activation.

Gene ontology analysis indicated significant overexpression of proteins involved in ribosome biogenesis, immune response, extracellular matrix (ECM) remodeling, and wound healing. Ribosome biogenesis is considered an important parameter for cell growth and division^66^; the overexpression of ribosome biogenesis proteins is related to mitochondrial gene expression, ECM and migratory processes, and polarization of macrophages toward an M2 phenotype.^67^ Considering immune response, M2 polarization is involved in immune regulation, inhibition of inflammation, and tissue repair and damage^68^; eventually, ECM remodelling and healing processes are also associated with M2 polarization.^69^

Overall, proteomic analysis indicates M2 polarization of microglia cells in the presence of Dox-CMC-*h*-BNs, while results of apoptosis and *K*_i_-67 expression suggest a therapeutic response against glioblastoma cells. M2 activation presents a promising avenue for anticancer therapy due to its potential to modulate the immune response, target tumor-associated macrophages, facilitate tissue remodelling and angiogenesis, and promote antitumor immunity via programmed cell death protein 1 (PD-1)/programmed cell death ligand 1 (PD-L1) interaction within the tumor microenvironment.^70^ It is important to note that exploiting M2 activation in anticancer therapy requires careful consideration and control to avoid potential drawbacks, such as excessive immunosuppression or increase of tumor-promoting processes. Further research is needed to better understand the intricate interactions among microglia, tumor cells, and the tumor microenvironment to optimize the therapeutic potential of M2 activation in the context of cancer treatment.

## 5 Conclusions

Nanomaterial-based drug delivery systems, such as *h*-BNs, offer promising strategies to enhance tumor targeting and drug delivery. The use of cell membrane-based strategies allows for natural interaction with biological systems, improved targeting effects, and increased biocompatibility. The successful coating of *h*-BNs with cell membranes was confirmed through various analyses including TGA, XPS, and western blotting. Therapeutic efficiency on glioblastoma cells was confirmed following treatment with Dox-CMC-*h*-BNs, while proteomics revealed significant modulation of proteins associated with the M2 polarization of microglia cells. While M2 activation shows potential for anticancer therapy by modulating the tumor microenvironment, further research is needed to optimize its therapeutic potential in cancer treatment, through *in vivo* preclinical testing confirming and validating our preliminary, yet promising, data.

## Data Availability

Proteomics data are available via ProteomeXchange with identifier PXD043557 [Reviewer account details: Username: reviewer_pxd043557@ebi.ac.uk; Password: e0XXdbdS]. All other data are available from the Corresponding Authors upon reasonable request.

## Supporting Information Available

Standard curves for doxorubicin concentration determination; EDS data; long-term stability studies; XPS wide-scan spectra; representative flow cytometry plots for cellular up-take analysis; representative 3D confocal rendering showing nanoparticle internalization; confocal Raman imaging of untreated cell cultures; representative confocal imaging of U87-MG cells stained for detection of *K*_i_-67 marker after 24 and 72 h of treatment; representative flow cytometry plots of apoptosis investigation; further proteomics data (PDF)
