# Supplementary material for: Microglia Polarization and Antiglioma Effects Fostered by Dual Cell Membrane-Coated Doxorubicin-Loaded Hexagonal Boron Nitride Nanoflakes
Source: ACS Appl Mater Interfaces. 2023 Dec 5;15(50):58260–73. doi: 10.1021/acsami.3c17097 (PMC10739601; doi:10.1021/acsami.3c17097)
Supplement: Supplementary file 1 — am3c17097_si_001.pdf [file am3c17097_si_001.pdf]

Supporting Information

Microglia polarization and anti-glioma effects fostered by dual cell membrane-coated doxorubicin-loaded hexagonal boron nitride nanoflakes

Özlem Şen[1,], Melis Emanet[1], Martina Mazzuferi[1,2], Martina Bartolucci[3], Federico Catalano[4], Mirko Prato[5], Stefania Moscato[6], Attilio Marino[1], Daniele De Pasquale[1], Giammarino Pugliese[7], Francesco Bonaccorso[8,9], Vittorio Pellegrini[8,9], Antonio Esau Del Rio Castillo[8], Andrea Petretto[3], Gianni Ciofani[1,*]*

[1]Istituto Italiano di Tecnologia, Smart Bio-Interfaces, Viale Rinaldo Piaggio 34, 56025 Pontedera (Pisa), Italy

[2]Politecnico di Torino, Department of Mechanical & Aerospace Engineering, Corso Duca degli Abruzzi 24, 10129 Torino, Italy

[3]IRCCS Istituto Giannina Gaslini, Core Facilities-Clinical Proteomics and Metabolomics, Via Gerolamo Gaslini 5, 16147 Genova, Italy

[4]Istituto Italiano di Tecnologia, Electron Microscopy Facility, Via Morego 30, 16163 Genova, Italy

[5]Istituto Italiano di Tecnologia, Materials Characterization Facility, Via Morego 30, 16163 Genova, Italy

[6]University of Pisa, Department of Clinical and Experimental Medicine, Via Roma 55, 56126 Pisa, Italy

[7]Istituto Italiano di Tecnologia, Nanochemistry, Via Morego 30, 16163 Genova, Italy

[8]BeDimensional SPA, Lungotorrente Secca 30R, 16163 Genova, Italy

[9] Istituto Italiano di Tecnologia, Graphene Labs, Via Morego 30, 16163 Genova, Italy

*Corresponding Authors: ozlem.sen@iit.it; gianni.ciofani@iit.it

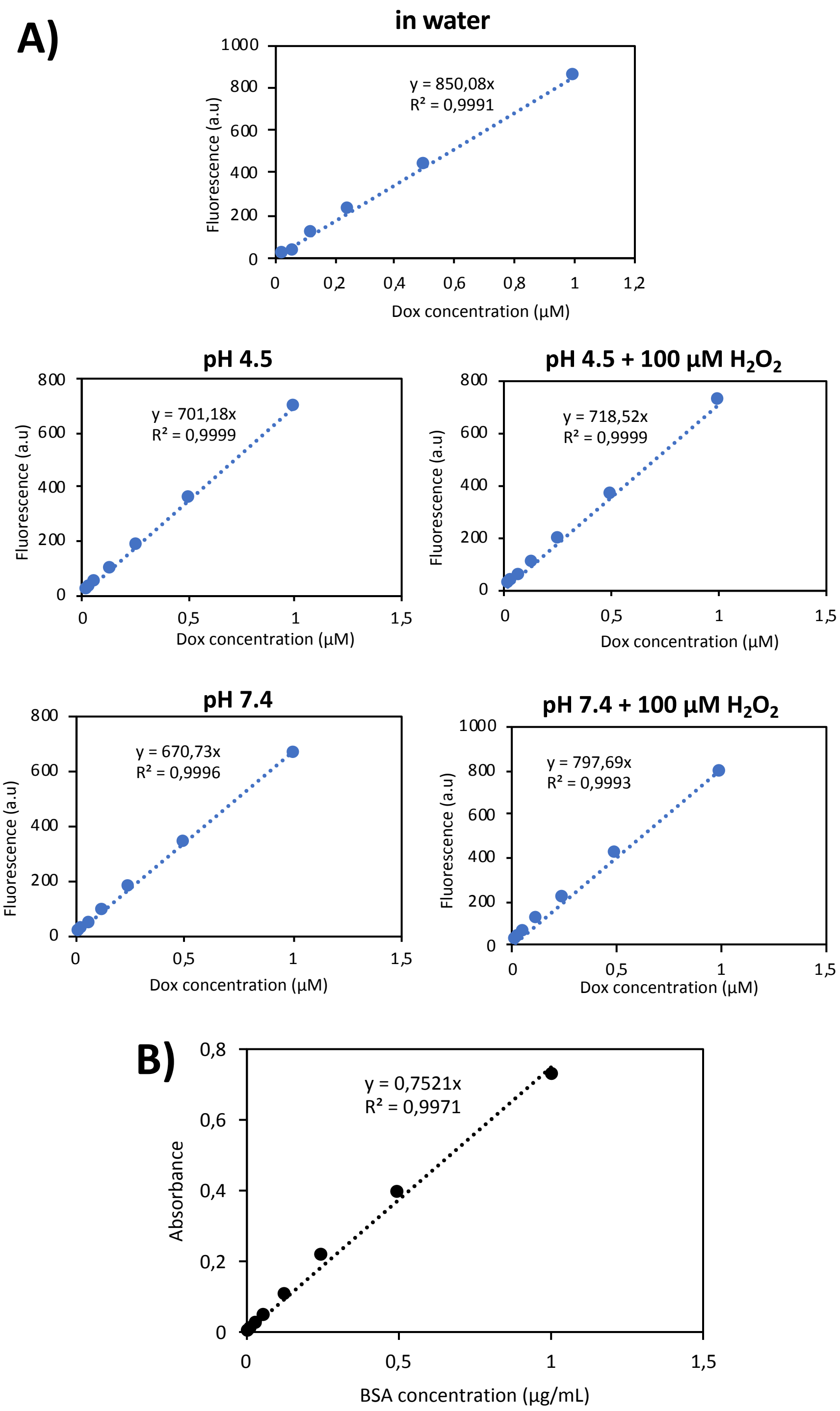

Figure S1. Standard curves for **A)** Dox detection in different media (fluorescence), and **B)** bovine serum albumin (BSA) for BCA analysis (absorbance).

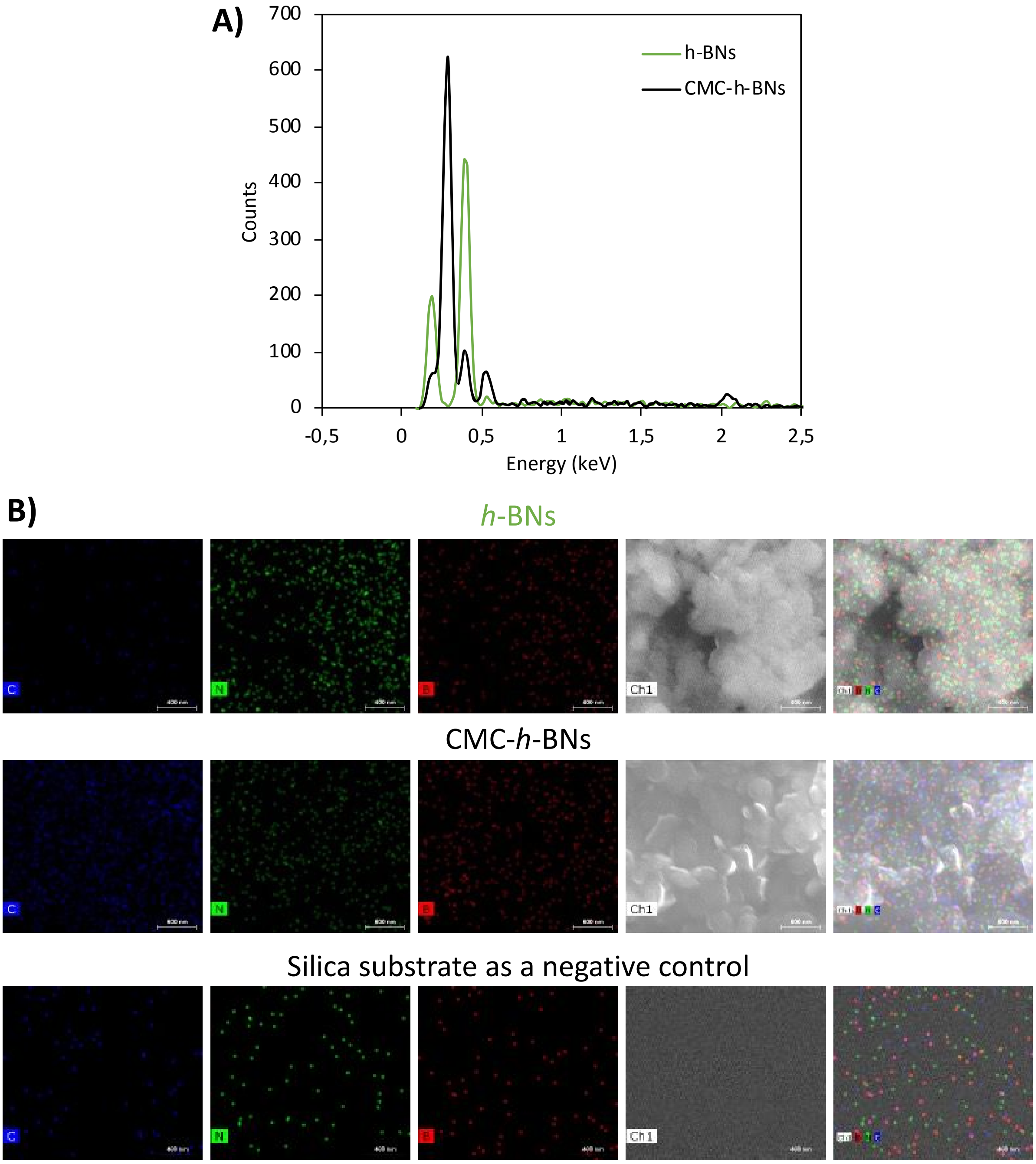

Figure S2. EDS **A)** spectrum and **B)** pseudo-colored elemental maps for B, N, C, O, and P in *h*-BN and CMC-*h*-BN samples. Elemental map of silica substrate is reported as background.

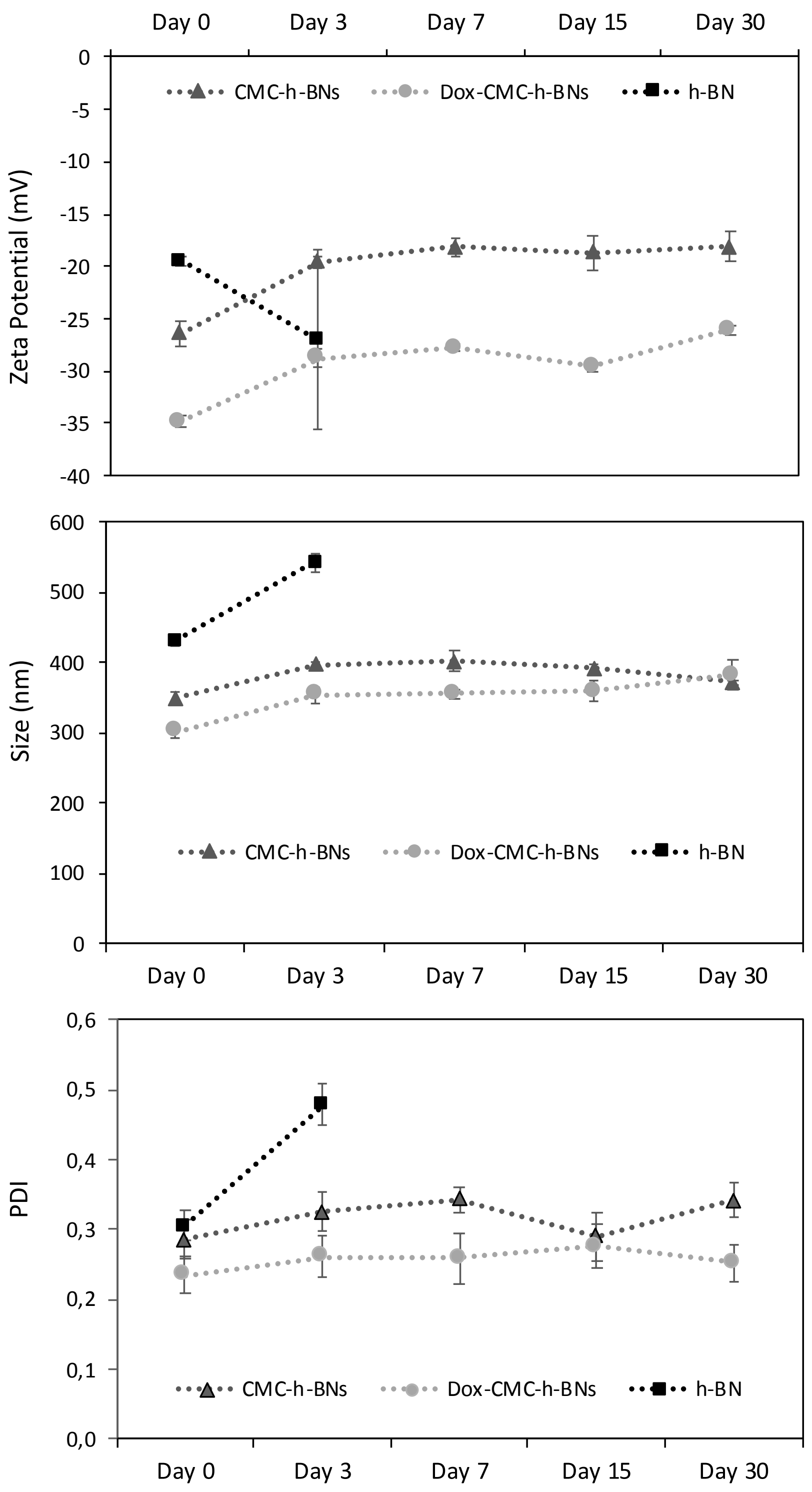

Figure S3. Long-term stability studies for *h*-BN, CMC-*h*-BN, and Dox-CMC-*h*-BN aqueous dispersions at 4°C.

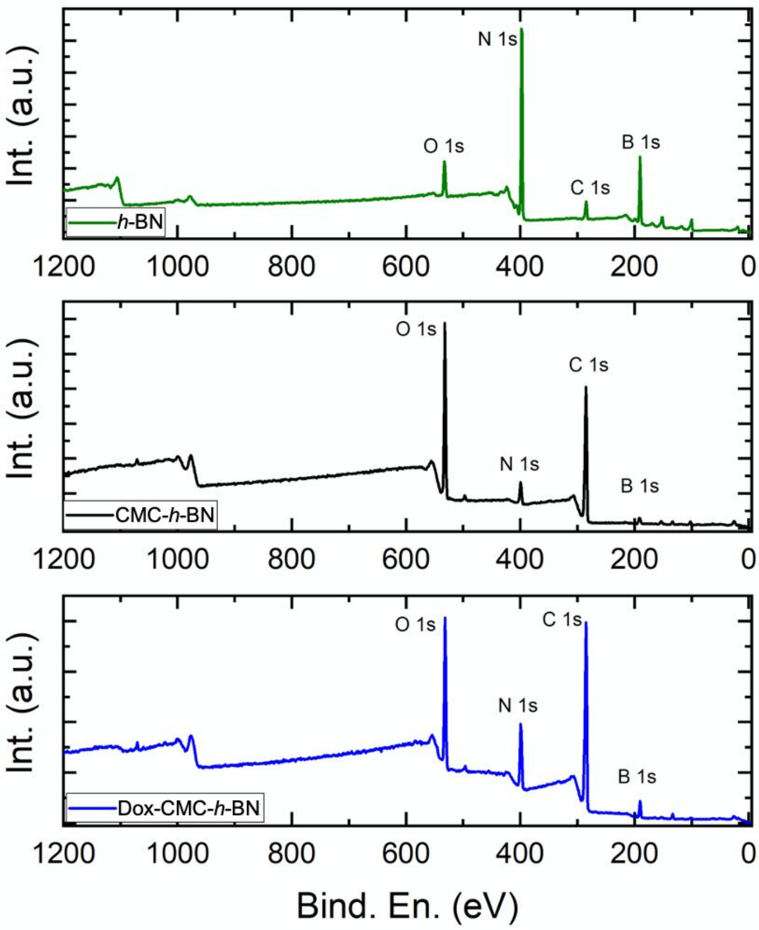

Figure S4. XPS wide-scan spectra of *h*-BN, CMC-*h*-BN, and Dox-CMC-*h*-BN samples.

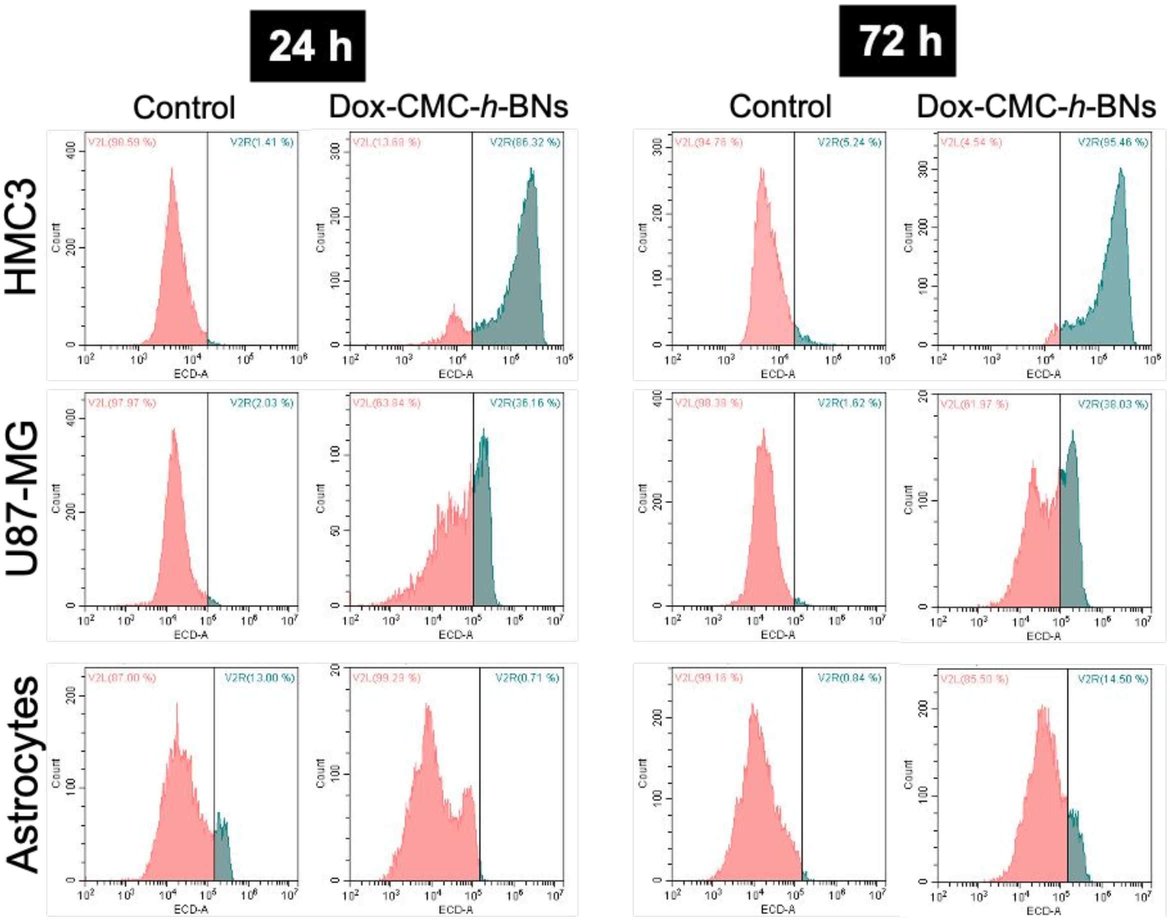

Figure S5. Representative flow cytometry plots for analysis of Dox-CMC-*h*-BN internalization after 24 and 72 h of incubation.

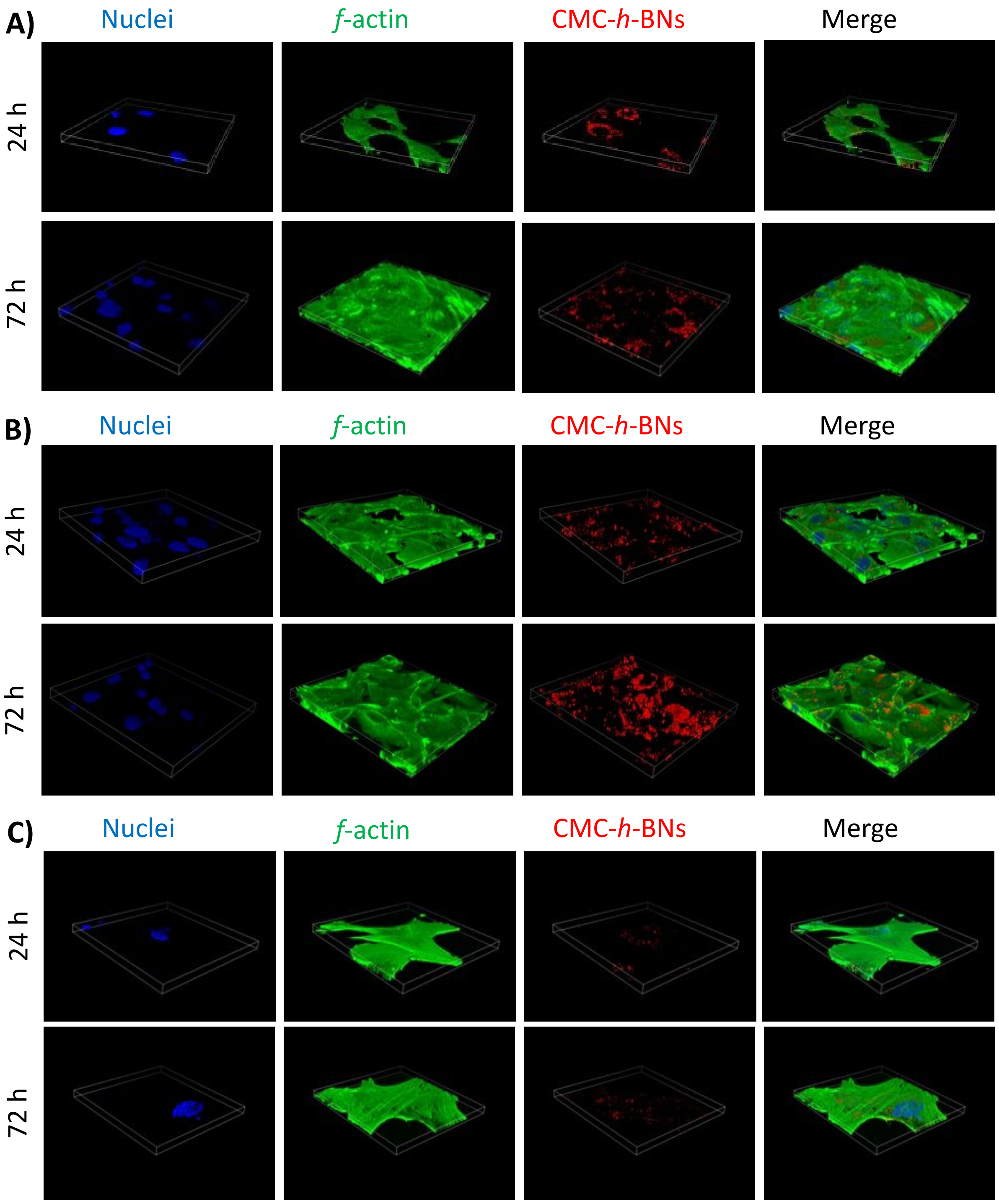

Figure S6. Representative 3D confocal rendering of **A)** HMC3, **B)** U87-MG, and **C)** astrocyte cultures showing nanoparticle internalization. Scan area size is: x = 124 µm, y = 124 µm, z = 8 µm (for HMC3); x = 124 µm, y = 124 µm, z = 9 µm (for U87-MG); x = 124 µm, y = 124 µm, z = 7 µm (for astrocytes).

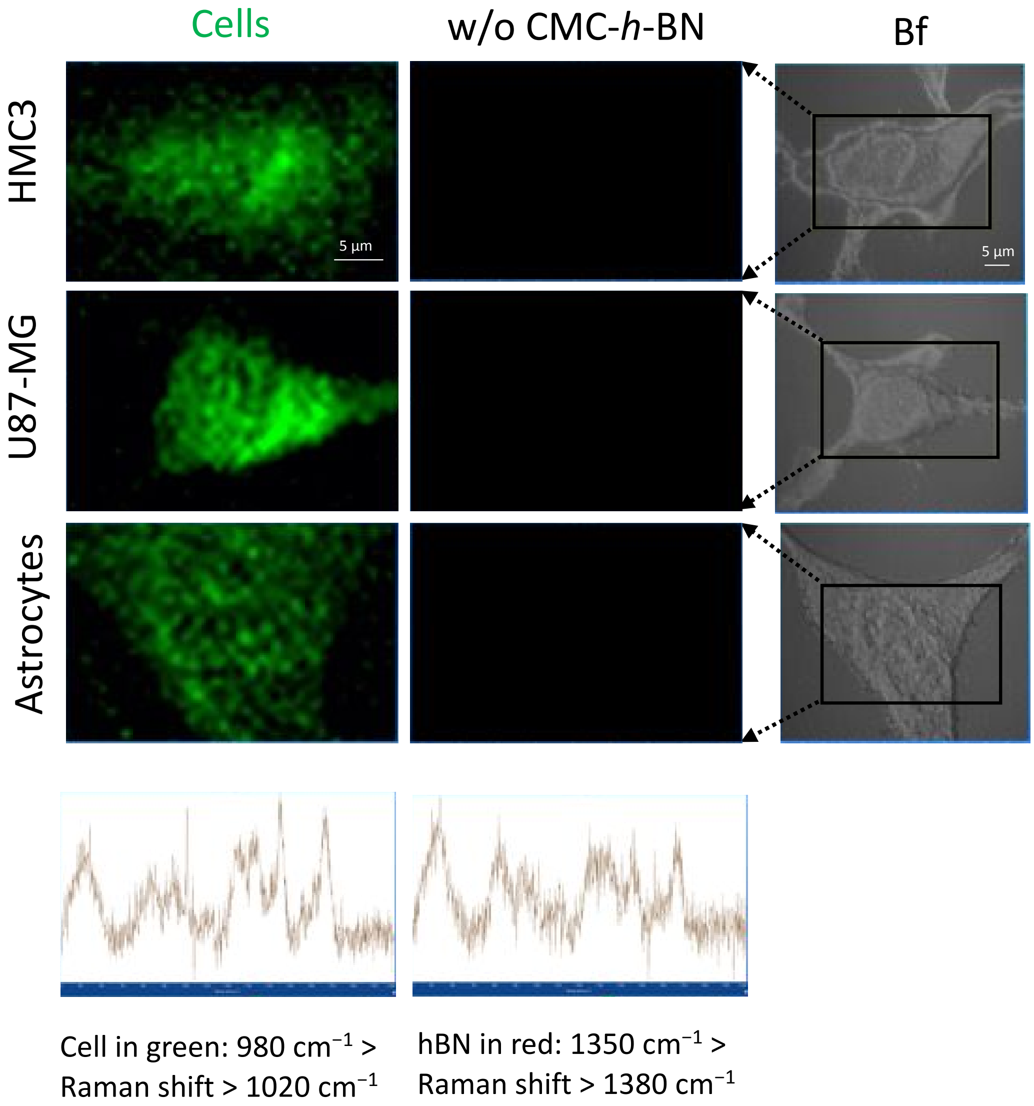

Figure S7. Confocal Raman imaging of untreated HMC3, U87-MG, and astrocytes. Representative Raman spectra of cells and *h*-BNs are also shown. Signal maps were obtained according to the signal of phenylalanine (in green, Raman shift range: 980−1020 cm^{-1}), and of *h*-BNs (in red, Raman shift range: 1350−1380 cm^{-1}).

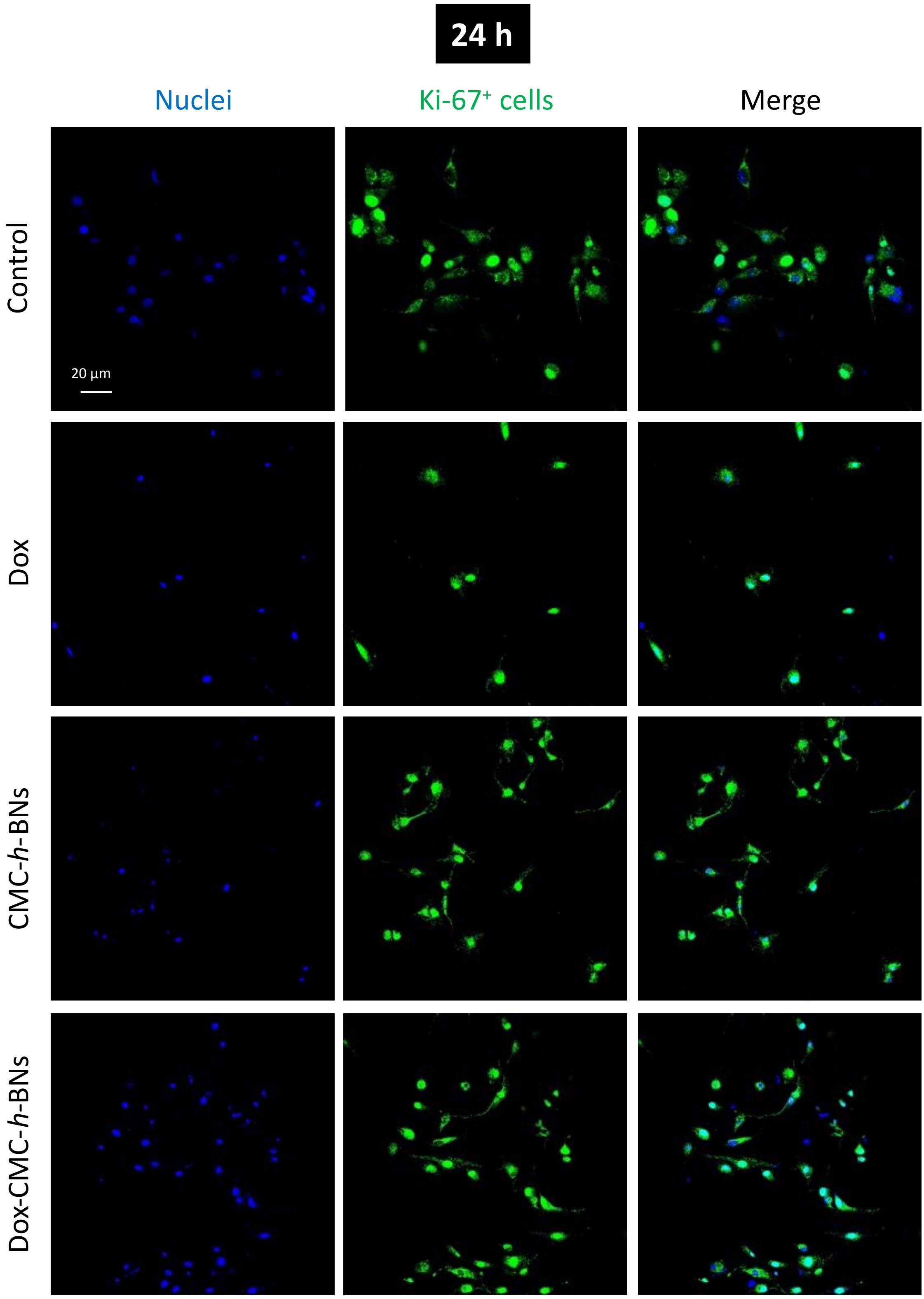

Figure S8. Representative confocal imaging of U87-MG cells stained for detection of Ki-67 marker after 24 h of treatment.

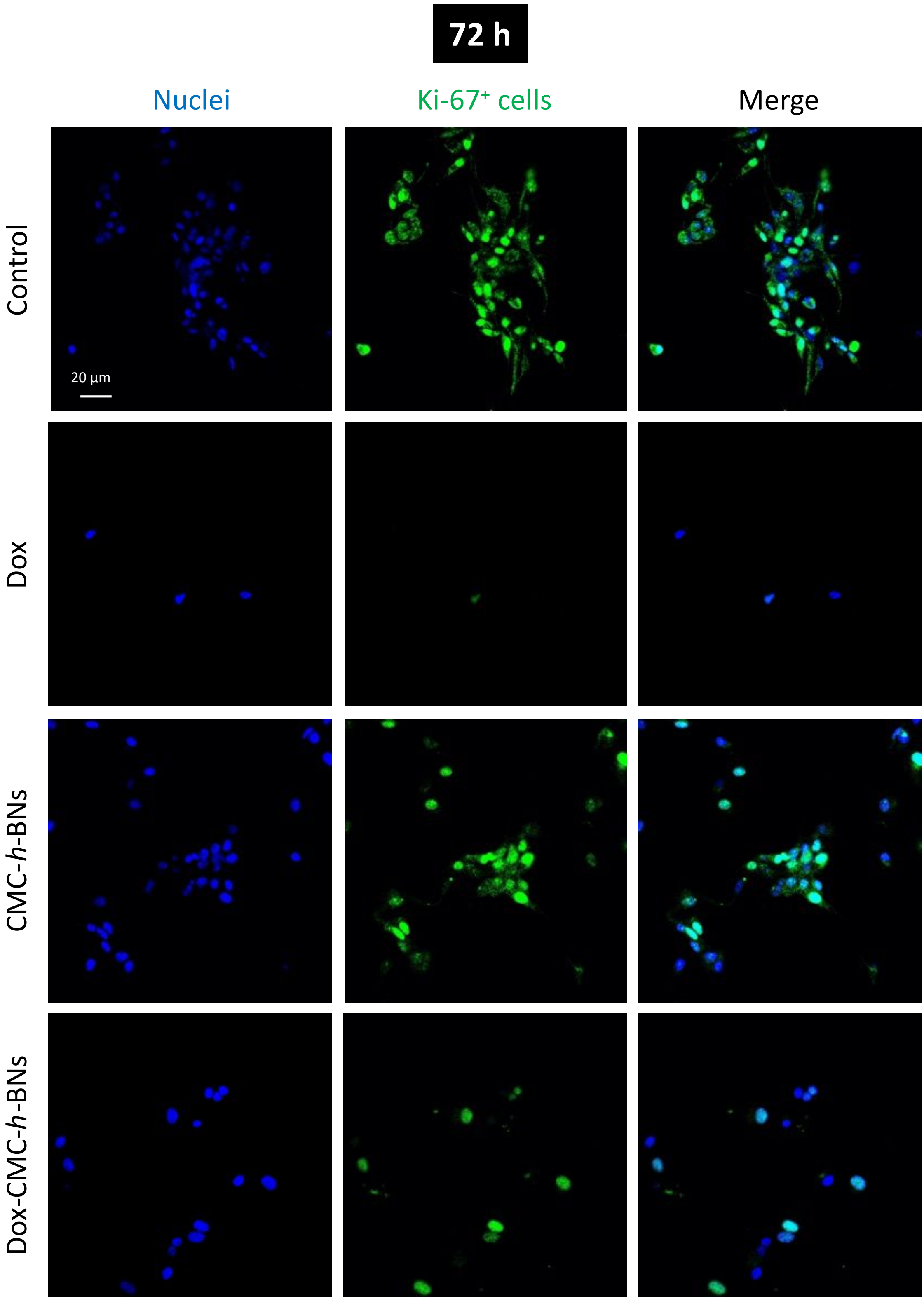

Figure S9. Representative confocal imaging of U87-MG cells stained for detection of Ki-67 marker after 72 h of treatment.

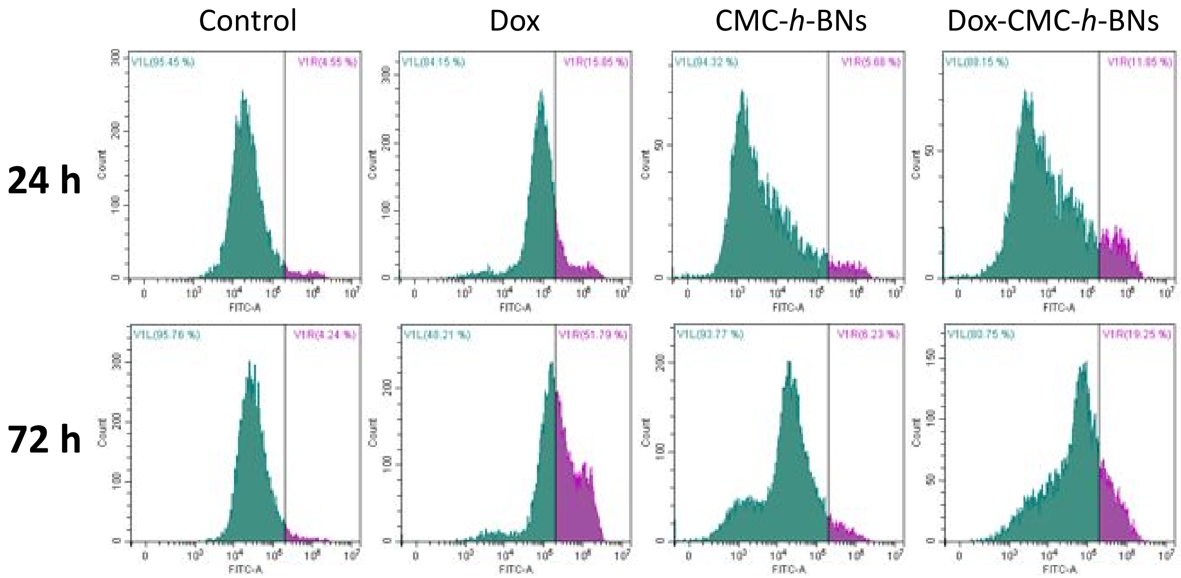

Figure S10. Representative flow cytometry plots of apoptosis investigation on U87-MG cells treated with Dox, CMC-*h*-BNs, and Dox-CMC-*h*-BNs for 24 and 72 h; control cultures are reported as well.

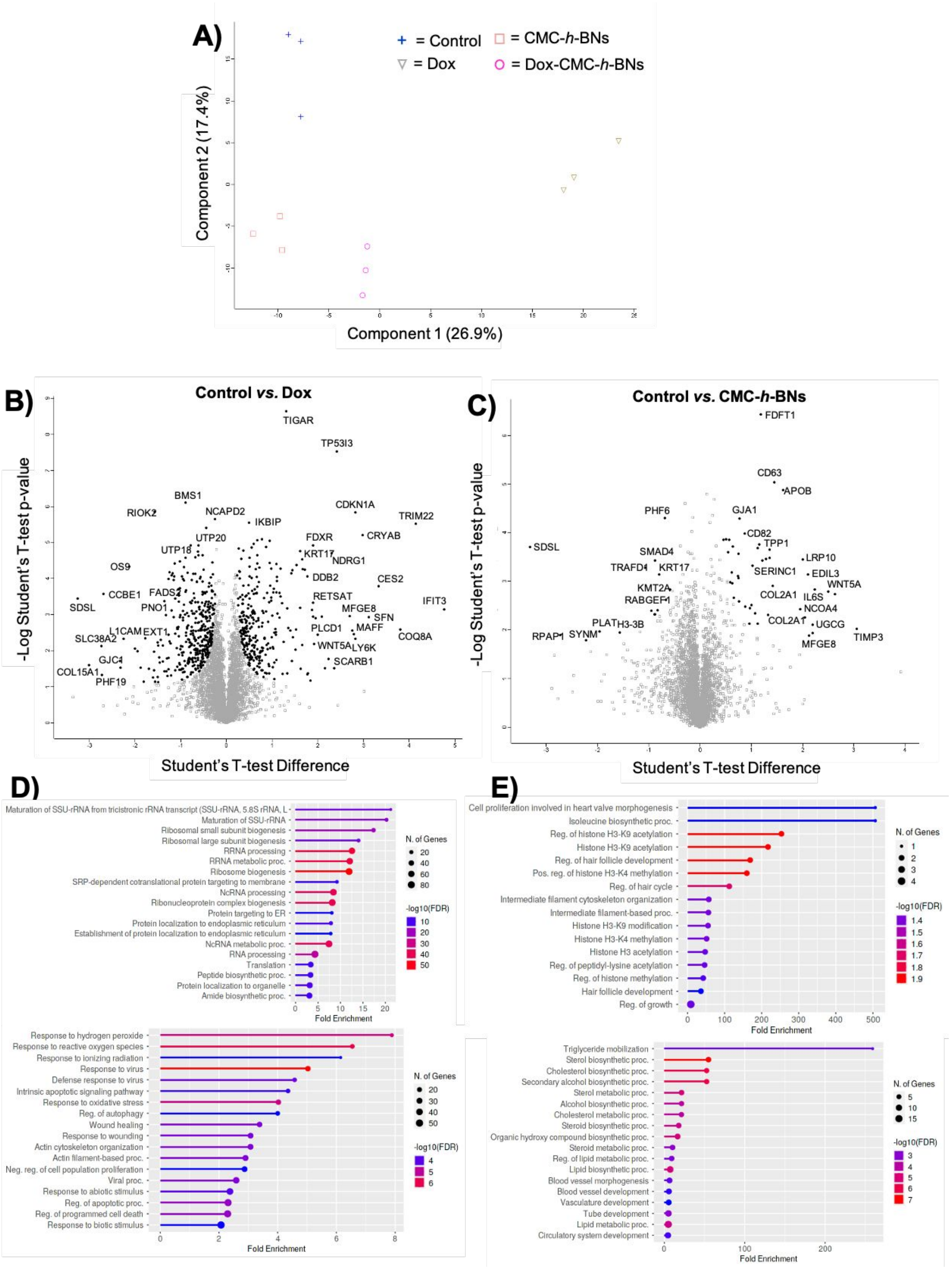

Figure S11. Proteomic investigation. **A)** Principal component analysis (PCA) for 3 experiments in each experimental class. Volcano plots showing over- and under-expression of proteins in **B)** Dox-treated and **C)** CMC-*h*-BNs-treated HMC3 cells with respect to control cultures. Complete list of the gene ontology terms related to the **D)** Dox, and **E)** CMC-*h*-BNs experimental classes.
